# Concatenation of paired-end reads improves taxonomic classification of amplicons for profiling microbial communities

**DOI:** 10.1186/s12859-021-04410-2

**Published:** 2021-10-12

**Authors:** Daniel P. Dacey, Frédéric J. J. Chain

**Affiliations:** Department of Biological Sciences, University of Massachusetts Lowell, Lowell, MA USA

**Keywords:** 16S, Joining, QIIME 2, DADA2, Mock, Taxonomic assignment, V1–3, V3–4, V4

## Abstract

**Background:**

Taxonomic classification of genetic markers for microbiome analysis is affected by the numerous choices made from sample preparation to bioinformatics analysis. Paired-end read merging is routinely used to capture the entire amplicon sequence when the read ends overlap. However, the exclusion of unmerged reads from further analysis can result in underestimating the diversity in the sequenced microbial community and is influenced by bioinformatic processes such as read trimming and the choice of reference database. A potential solution to overcome this is to concatenate (join) reads that do not overlap and keep them for taxonomic classification. The use of concatenated reads can outperform taxonomic recovery from single-end reads, but it remains unclear how their performance compares to merged reads. Using various sequenced mock communities with different amplicons, read length, read depth, taxonomic composition, and sequence quality, we tested how merging and concatenating reads performed for genus recall and precision in bioinformatic pipelines combining different parameters for read trimming and taxonomic classification using different reference databases.

**Results:**

The addition of concatenated reads to merged reads always increased pipeline performance. The top two performing pipelines both included read concatenation, with variable strengths depending on the mock community. The pipeline that combined merged and concatenated reads that were quality-trimmed performed best for mock communities with larger amplicons and higher average quality sequences. The pipeline that used length-trimmed concatenated reads outperformed quality trimming in mock communities with lower quality sequences but lost a significant amount of input sequences for taxonomic classification during processing. Genus level classification was more accurate using the SILVA reference database compared to Greengenes.

**Conclusions:**

Merged sequences with the addition of concatenated sequences that were unable to be merged increased performance of taxonomic classifications. This was especially beneficial in mock communities with larger amplicons. We have shown for the first time, using an in-depth comparison of pipelines containing merged vs concatenated reads combined with different trimming parameters and reference databases, the potential advantages of concatenating sequences in improving resolution in microbiome investigations.

**Supplementary Information:**

The online version contains supplementary material available at 10.1186/s12859-021-04410-2.

## Background

Microbial community profiling using 16S rRNA gene amplicon sequence analysis has become a powerful procedure to characterize microbiomes, but there exists enormous variation in the choice of bioinformatics tools and pipelines that can lead to different taxonomic assignments with the same dataset [1–5]. The performance of different analytical approaches is affected by numerous factors including amplicon target [6], targeted community [7–9], DNA extraction methods [10], PCR polymerase [11], sequencing platform [12], sequence quality [13], index hopping [13, 14], substitution errors [15], and read length [16]. Post-PCR paired-end sequence processing decisions such as how to remove low quality base pairs and merge reads can also influence taxonomies reported.

Low quality bases are usually removed by trimming all sequences by the same user-specified length (length trimming) or by an average quality score per individual sequence (quality trimming). Length trimming results in a FASTQ file with all sequences trimmed to the same exact size, whereas trimming by a quality threshold allows for each sequence to be evaluated independently, leaving sequences within a file to be trimmed at different lengths. Length trimming has the advantage that the user determines where sequences are trimmed and can ensure sufficient overlap for merging paired reads, but it runs the risk of removing high quality base pairs. Trimming 16S rRNA gene sequences by length might also be preferable over quality trimming for unbiased clustering operational taxonomic units (OTUs) [17], and it reduces the number of amplicon sequence variants (ASVs) by eliminating length variation. In both cases though, trimming might cause a loss of informative base pairs necessary for merging paired reads or important for distinguishing between closely related taxa.

**Fig. 1:**
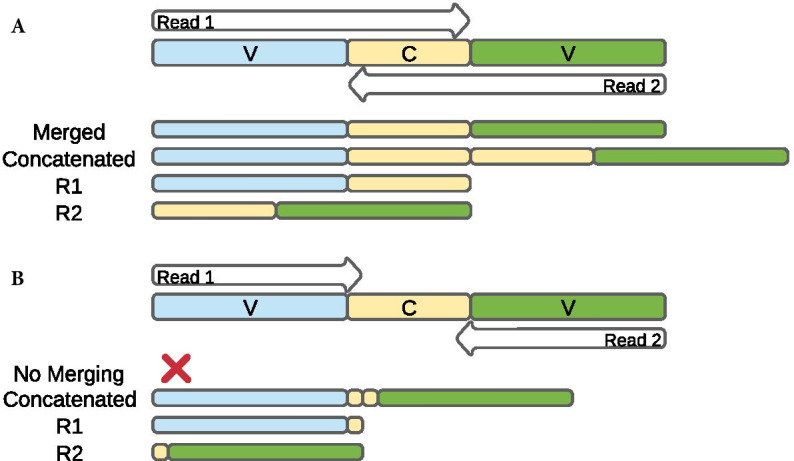
Representation of merged and fully concatenated reads in instances where paired-end sequences **A** do overlap and **B** do not overlap. Forward reads (R1) and reverse reads (R2) cover variable regions (V) that can include parts of conserved regions (**C**)

Paired-end read merging is often performed to reconstitute the full amplicon length from overlapping paired reads. This merging process can be hindered if the designed primers target amplicons longer than both read pairs combined, if organisms have amplicon sequences that are longer than the read lengths, and if sequences with poor quality bases, in particular at the ends of reads and in the reverse (R2) reads, result in trimming reads to short lengths that prevent read overlap [18]. In most cases, reads that fail to merge are excluded from taxonomic assignment, potentially reducing estimates of microbiome diversity. To remedy this data loss of sequences from merging, it is common to only use the higher quality forward (R1) reads for taxonomic assignment [19–22]. Though more sequences are retained, the length of the sequence per amplicon is shorter than if the R2 reads were included, which may in turn limit taxonomic resolution and accuracy. An alternative option is to concatenate (join) the paired reads together without requiring sequence overlap, which can introduce redundant data in the middle of the sequence if reads overlap but retains both reads for taxonomic assignment (Fig. 1). Tools developed to address the problem of non-overlapping reads have found that a combination of merged-reads and single-end reads [18] or concatenated (joined) sequences [17, 23] lead to improved taxonomic resolution compared to single-end pipelines, but these studies have all been tested on OTUs rather than ASVs. OTUs are reads clustered by a sequence identity threshold [24, 25], which is helpful for grouping similar reads together for estimating diversity, especially when taxonomic assignment is not possible due to a lack of reference sequences. But OTUs can both inflate diversity estimates and reduce taxonomic resolution, while limiting the ability to track OTUs across studies [26, 27] as their identifiers are unique to each sequencing output. ASVs largely overcome these limitations, allowing analysis at the single nucleotide level to increase taxonomic resolution over OTUs and improve reproducibility, thus becoming the preferred approach to 16S rRNA gene investigations [25, 28, 29]. It is important to test out the performance of read concatenation versus merged reads with ASVs, which to our knowledge has not yet been evaluated.

**Fig. 2:**
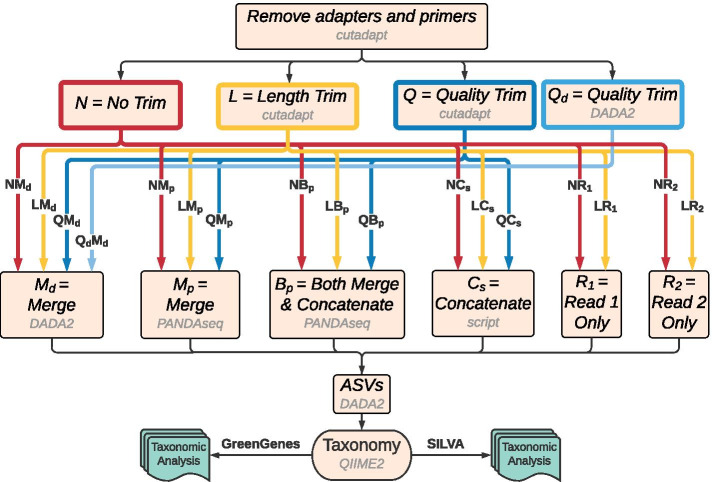
16S rRNA gene sequence processing steps for each of the 17 pipelines. All raw sequences began with identical adapter/primer removal by *cutadapt*. Paired-end sequences were then fed into each of the four trimming procedures: no trimming (N; red), length trimming by *cutadapt* (L; orange), quality trimming by *cutadapt* (Q; dark blue), and quality trimming by *DADA2* (Qd; light blue). Non-trimmed paired-end sequences were piped directly into the merging/concatenating tools after primers were removed. Sequences designated for trimming were merged and/or concatenated after trimming by *cutadapt* or DADA2. Paired-end sequences designated for merging by DADA2 (Md) were first passed through DADA2’s quality filter and denoising procedures before being merged and chimeras removed. ASV curation immediately followed. Sequences run through the only Qd pipeline had all steps from trimming to ASV designations performed within DADA2. The majority of pipelines included length and quality trimming of paired-end sequences in *cutadapt* that were then piped into PANDAseq for merging (Mp) or merging and concatenating of those sequences unable to merge (Bp). Full concatenation (Cs) of paired-end sequences was done with a custom script after *cutadapt* trimming. Mp, Bp, and Cs sequences were fed into DADA2 as single-end where quality filtering, denoising, chimera removal, and ASV curation occurred. Non-trimmed single-end (NR1, NR2) and *cutadapt* length-trimmed single-end (LR1 and LR2) sequences did not go through a merging or concatenating procedure, but were submitted in the same manner to DADA2 as Mp, Bp, and Cs. Post-DADA2, taxonomic classification of ASVs was identical for all pipelines. ASVs were fed into QIIME 2’s *feature-classifier classify-sklearn* tool which aligned ASVs to one of two designated reference databases separately, Greengenes and SILVA. These reference database aligned files were then merged with the appropriate metadata files before taxonomic analysis in R

Additional choices made in paired-end sequence processing pipelines that can affect the taxonomic assignment include the sequence abundance threshold [30], reference database [31], and amplicon length [6], but their influence on the utility of concatenated reads is unknown. Here, we conducted an extensive assessment of 17 different pipelines (Fig. 2) for processing 16S rRNA gene sequences from ten mock communities (Table 1) to determine how genus-level classification accuracy is affected by (1) ASV abundance thresholds, (2) reference databases, (3) length and quality trimming, (4) merging and concatenating, and (5) single-end reads. All reads were run through DADA2 within QIIME 2 [32], and ASVs were taxonomically classified using the Greengenes [33] and SILVA [34] reference databases.

**Table 1:** Bacterial mock communities used in pipeline analyses

Study ID^a^	Amplicon	Mock type	No. isolates^b^	No. Genera	No. read pairs	Read length	R1 trimmed-length	R2 trimmed-length	Avg. Q R1	Avg. Q R2
A1	V1-3	Balanced	19	19	950,404	301	301	225	34	29
C1	V4	Unbalanced	27	11	8,161,940	251	235	217	33	29
G1	V4	Balanced	20	17	1,426,252	301	301	258	34	32
G2	V4	Balanced	20	17	828,788	301	299	207	33	27
G3	V4	Unbalanced	20	17	1,346,668	301	301	229	34	31
G4	V4	Unbalanced	20	17	832,756	301	299	209	33	27
S1	V4	Balanced	49	37	3,722,660	250	240	220	36	36
S2	V4	Unbalanced	49	37	2,103,960	250	240	220	36	35
S3	V3-4	Balanced	49	37	357,444	250	240	220	35	35
S4	V3-4	Balanced	49	37	1,644,752	250	240	220	36	35

^a^Sequence information can be found in a separate table (Additional file 3: Table S1)

^b^Mock community composition can be found in a separate table (Additional file 3: Table S2)

## Methods

### Data

The 16S rRNA gene sequences used in this study came from ten bacterial mock communities (herein referred to as ‘mocks’) from four studies and are labelled C1 [35], G1-G4 [36], S1-S4 [37], and A1 [38]. These sequenced mocks varied in 16S rRNA gene amplicon region, taxonomic composition, read depth, read length, and sequence quality (Table 1). The raw FASTQ files were already demultiplexed with sequencing adapters removed. The number of samples, primer sequences, and accessions for each mock community are available (Additional file 3: Table S1), as are the bacterial makeup of each (Additional file 3: Table S2).

### Sequence processing

The 16S rRNA gene sequences from each mock community were processed with 17 bioinformatic pipelines to test the joint effects of different combinations of sequence trimming, merging, concatenating, single vs paired reads, and reference databases on taxonomic assignment (Fig. 2; Table 2). All bioinformatic processing was performed on The Massachusetts Green High Performance Computing Cluster. Depending on the pipeline, read trimming was performed with *cutadapt version 2.9* [39] or with DADA2 [35], merging was performed using *PANDAseq version 2.11* [40] or DADA2, concatenation was performed using PANDAseq or a custom Python script, and reads were assigned taxonomy using either Greengenes *version 13_8* or SILVA *version 132* within the tool *Quantitative Insights into Microbial Ecology version 2* (QIIME 2) *version 2020.6* [32]. DADA2 was used as a plugin within QIIME 2.

**Table 2:** Layout of sequence processing parameters per pipeline

Pipeline	Cutadapt trimming	Merging/Concatenating	DADA2 processing^h^
NMd	None	Merged by DADA2^d^	Paired-end
LMd	Length-trimmed	Merged by DADA2	Paired-end
QMd	Quality-trimmed	Merged by DADA2	Paired-end
QdMd	None – Quality-trimmed by DADA2^a^	Merged by DADA2	Paired-end
NMp	None	Merged^e^	Single-end
LMp	Length-trimmed^b^	Merged	Single-end
QMp	Quality-trimmed^c^	Merged	Single-end
NBp	None	Merged & Concatenated^f^	Single-end
LBp	Length-trimmed	Merged & Concatenated	Single-end
QBp	Quality-trimmed	Merged & Concatenated	Single-end
NCs	None	All Concatenated^g^	Single-end
LCs	Length-trimmed	All Concatenated	Single-end
QCs	Quality-trimmed	All Concatenated	Single-end
NR1	None	N/A	Single-end
LR1	Quality-trimmed	N/A	Single-end
NR2	None	N/A	Single-end
LR2	Quality-trimmed	N/A	Single-end

Default parameters were used for each tool unless otherwise specified. The first step for all pipelines was to remove primers with *cutadapt*. Paired-end reads were merged by DADA2, or alternatively merged, concatenated, or merged and concatenated prior to DADA2 processing (resulting in single-end reads) or treated separately without merging or concatenation. After DADA2 processing, taxonomic classification of all sequences was performed by aligning to both the Greengenes and SILVA reference databases separately

^a^DADA2 performed the trimming, with trunc-q set to 20

^b^Length trimmed: all sequences were trimmed to the same length based on the mean length where the average base quality dropped below a Q-score of 20

^c^Quality trimmed: sequences were individually trimmed based on a PHRED score threshold of 20

^d^Merged by DADA2: forward and reverse reads merged by DADA2 with default parameters with a minimum 20 bp overlap

^e^Merged: forward and reverse reads merged by PANDAseq with a minimum 20 bp overlap

^f^Merged and concatenated: after merging with PANDAseq, sequences unable to be merged were concatenated with PANDAseq and added to the merged sequence file

^g^All concatenated: forward and reverse read pairs were joined together after reverse complementing R2. No merging was performed

^h^DADA2 processing: pipelines with merging and/or concatenating before this step, or that contained just the forward or reverse reads, were processed as single-end. Pipelines that had DADA2 performing the merging were processed as paired-end

The naming schema for the 17 pipelines is based on the type of trimming (none, length, or quality), merging and/or concatenating of paired reads, what tool was used to perform the merging, and whether the pipeline contained both paired-end reads or just one of the pair (see Fig. 2). Pipelines having paired-end sequences with no further trimming after primer removal were designated as not trimmed (N). If trimming was performed then it was done so in three different manners: length-trimmed by *cutadapt* (L), quality-trimmed by *cutadapt* (Q), or quality-trimmed by DADA2 (Qd). Merging and/or concatenating of paired-end reads followed one of four fashions: merging by PANDAseq (Mp); merging by DADA2 (Md); merging by PANDAseq combined with concatenating with PANDAseq those reads unable to be merged (both, Bp); and fully concatenating all read pairs with a custom Python script (Cs). Concatenation is the process of appending the forward and reverse reads together (after reverse complementing the reverse read) as a single joined sequence. The names of the 13 paired-end read pipelines are NMp, NBp, LMp, LBp, QMp, QBp, NMd, LMd, QMd, QdMd, NCs, LCs, and QCs. The single-end pipelines used either just the forward reads (R1) or just the reverse reads (R2). These pipelines follow the same nomenclature as paired-end pipelines, having either no trimming (N) or length trimming (L), but merging and/or concatenating was not applicable to single-end pipelines. Quality trimming on single-end reads was also not performed as our investigation revealed paired-end length-trimmed pipelines to outperform their quality-trimmed counterparts (Table 3). The four single-end read pipelines are named NR1, LR1, NR2, and LR2. Below, we describe in more detail the steps taken for the different pipelines including the parameters used for read trimming (N, L, Q), paired-end read preparation (Mp, Md, Bp, Cs), and taxonomic assignment (using Greengenes and SILVA reference databases).

**Table 3:** Paired-end pipeline mean metrics across all mocks.*

Pipeline	Precision mean	Recall mean	F-Measure mean
LCs	0.957	0.864	0.897
QBp	0.916	0.852	0.874
LMd	0.938	0.832	0.871
LBp	0.953	0.827	0.858
QMd	0.953	0.803	0.854
LMp	0.940	0.818	0.847
QMp	0.915	0.799	0.831
NBp	0.859	0.809	0.830
QCs	0.962	0.753	0.821
NMp	0.854	0.797	0.820
NMd	0.959	0.694	0.766
NCs	0.960	0.670	0.731
QdMd	0.869	0.478	0.551

^*^Ordered by descending average F-measure using SILVA reference database and ASV abundance threshold cutoff of 0.01%

### Read trimming

Removal of primer sequences was performed with *cutadapt* using the primer sequences corresponding to each mock community (Additional file 3: Table S1). Primer detection and removal was performed starting at the 5’ end of all sequences in the forward and reverse FASTQ files. When trimming was performed, *cutadapt* first trimmed from 3’ ends and then removed primers from the 5’ ends. Default settings and a minimum sequence length of 1 were used. All pipelines had primer sequences removed.

Non-trimmed reads (NMp, NBp, NMd, NCs, NR1, and NR2) had only primer sequences removed with *cutadapt* and were otherwise full-length. Length-trimmed reads (LMp, LBp, LMd, LCs, LR1, and LR2) were trimmed with *cutadapt* to achieve equal read lengths for all reads of a library. This way, identical sequences will be assigned the same amplicon sequence variant (ASV) in DADA2 as described below. In contrast trimming reads by sequence quality has the chance to trim two identical sequences to different lengths, increasing the number of ASVs. To determine the trimming length, the raw FASTQ files of each mock community were submitted to QIIME 2 for generation of Phred quality score (Q-score) distribution plots (Additional file 1: Fig. S1). Mock communities that had multiple samples were combined so that each mock only had one Q-score plot associated with it. Moving in the 5’ to 3’ direction, the first occurrence of the median Q-score falling below 20 was the location chosen for length trimming (Table 1; Additional file 3: Table S1). For example, mock C1 met this threshold at base 235 out of 251 for R1 and at 217 out of 251 for R2, so reads were subsequently trimmed to these lengths. All base pairs were kept in instances where the Q-score never fell below 20 (*cutadapt* trim position = 0 retains all base pairs) except for mocks S1-4, in which R1 was trimmed to position 240 and R2 to position 220 to align with a previous study [29]. Quality-trimmed reads (QMp, QBp, QMd, QCs) were trimmed with *cutadapt* using a Q-score threshold of 20 from the 3’ end of both R1 and R2 reads, but for each individual read, resulting in variable read lengths within a library. Higher quality sequences within a library would retain more base pairs with quality trimming than they would when they are length-trimmed, as the latter trims an entire library at a specified position based on the average quality distribution. For the QdMd pipeline, sequences had primers removed with *cutadapt* first bring submitted to QIIME 2 where DADA2 performed quality trimming (default settings with a Q-score = 20) as opposed to *cutadapt*.

### Merging and concatenating

After trimming, paired-end reads were either merged (Mp or Md), merged and concatenated (Bp), only concatenated (Cs), or processed as single-end reads (R1 or R2). Merging of R1 and R2 reads (NMp, LMp, QMp) was performed with PANDAseq with default parameters, which assembles paired reads that have a minimum overlap of 20 base pairs (the default minimum overlap in DADA2) and discards unmerged reads. PANDAseq is software not included in QIIME 2 but was used here because it also allows concatenation of unmerged paired sequences, which appends R1 and R2 reads together in a single contiguous sequence and adds them to the merged reads file. This concatenating feature was chosen for merged and concatenated pipelines (NBp, LBp, QBp). Thus, the pipelines NBp, LBp, and QBp have all of the reads that NMp, LMp, and QMp have respectively, but with the addition of the concatenated read pairs. PANDAseq was unable to produce files of only concatenated sequences (without merging), so a Python script was used to reverse complement R2 reads and concatenate them to their corresponding R1 reads for fully concatenated sequence files (NCs, LCs, QCs). Because there was no minimum overlap requirement like merging, no sequence information was lost at this step, but repetitive bases are included if R1 and R2 reads overlap (Fig. 1). DADA2-merged pipelines (NMd, LMd, QMd, QdMd) were the only pipelines that used paired-end sequence information not processed by PANDAseq or the concatenating Python script, since the DADA2 QIIME 2 plugin did not offer read concatenation at the time of this study. After *cutadapt* processing, sequences from DADA2-merged pipelines were submitted to DADA2 as paired-end, where DADA2 performed all merging in addition to other quality control measures described below. Again, QdMd was the only pipeline where reads were trimmed (by quality) and merged within DADA2. Because NR1, LR1, NR2, and LR2 used only single-end sequences, they were not subject to any merging or concatenating.

### DADA2 for ASV generation

All pipelines used DADA2 within QIIME 2 in one of two ways; single-end or paired-end. Reads that were merged and/or concatenated using PANDAseq or the concatenation script (NMp, NBp, LMp, LBp, QMp, QBp, NCs, LCs, QCs) were run through DADA2 as single-end with default parameters. DADA2 performed quality filtering (maxN = 0, truncQ = 2, rm.phix = TRUE and maxEE = 2), denoising, and chimera removal. Pipelines containing only R1 reads (NR1 and LR1) or R2 reads (NR2 and LR2) were also submitted to DADA2 in this way. For those pipelines where merging was performed in DADA2 (NMd, LMd, QMd, QdMd), sequences were submitted to DADA2 as paired-end. DADA2 performed quality filtering, quality trimming (only for QdMd), denoising, merging, and chimera removal on sequences with default parameters. DADA2 generated output read counts after each step (Additional file 3: Table S3) and assigned reads to ASVs that were then used for taxonomic assignment by aligning to a reference database.

### Taxonomic assignment

ASVs, which are unique DNA sequences, were assigned taxonomy separately using the SILVA and Greengenes (GG) reference databases, generating two taxonomic output files per mock/pipeline. Taxonomic assignment of ASVs was performed with the QIIME 2 plugin *feature-classifier classify-sklearn*. *sklearn* is a machine-learning-based classification method that requires trained classifiers of desired reference databases. QIIME 2 provides full-length 16S rRNA gene Naive Bayes trained SILVA and GG classifiers, which were used in this study, specifically SILVA 132 99% OTUs full-length sequences [41] and GG 13_8 99% OTUs full-length sequences [42]. The QIIME 2 *feature-classifier classify-sklearn* p-confidence value was set to the default of 0.7, a value thought to provide a good balance between recall and precision for 16S rRNA gene datasets [43]. The p-read-orientation setting within *feature-classifier classify-sklearn* specifies the direction in which the query sequence reads (ASVs) should be aligned against the reference, and this differed among mocks. Paired-end pipelines of mocks C1, G1-4, and S1-4 were aligned with p-read-orientation ‘same,’ as were mock A1’s single-end pipelines. Paired-end pipeline A1 and single-end pipelines of mocks C1, G1-4, and S1-4 were aligned with p-read-orientation ‘reverse.’

### Taxonomic analysis

R version 4.0.3 (2020–10-10) [44] was used for downstream analysis of taxonomic assignments. QIIME 2 outputs taxonomy assignments *from feature-classifier classify-sklearn* in BIOM format. R packages (phyloseq, stringr, dplyr, data.table, reshape2, ggplot2, gridExtra, VennDiagram, vegan, and gplots) were used for statistical calculations and for generating tables and figures. BIOM files consist of ASV counts, ASV taxonomic classifications, unique MD5 hash identifiers that link back to the original sequence, and sample metadata.

Taxonomic filtering was performed to focus on bacterial taxa and to adjust for variation in nomenclature in reference databases. ASVs having kingdom level classification other than “bacteria” were removed before analysis. There were 8 genera that were originally thought to be false positives due to the use of synonymous names that were actually true positives (Additional file 3: Table S4), so these taxa names were changed to match the naming schema of the mock communities. Some results with ambiguous taxonomic resolution were reclassified manually as ‘unknown’: when family level classification was the same as the genus level, and when additional numbers (that often related to uncultured strains) or words existed in the genus name that prevented pattern matching with the mock community dataset (UCG, NK, group, of, soil, clade, env, genus, group, candidatus, species, subsp, subgroup, subsec, marine, lineage, metagenome, mitochondria, R1, chloroplast, Incertae, strain numbers, and NA). The original unmodified genus names were compared to the modified genus names to ensure no genera were being lost due to these fixes in nomenclature. This nomenclature standardization process allowed direct comparisons of genus names between GG, SILVA, and each mock community.

### Pipeline performance measures

Pipeline performance was evaluated at the genus level using true positive (TP) counts, false positive (FP) counts, false negative (FN) counts, precision, recall, and F-measure. A TP was considered when a pipeline reported a genus name identical (or synonymous; Additional file 3: Table S4) to a genus name contained in the mock community (Additional file 3: Table S2). A FP was considered when a pipeline reported a genus not found in the mock community (misclassification or contamination). A FN was considered when a pipeline did not report a genus contained in the mock community, and thus was not observed. Counts of TP, FP, and FN were used to calculate precision, recall, and F-measure. Precision is the fraction of genera that are classified correctly. Recall is the fraction of expected genera that are classified. F-measure is the harmonic mean of precision and recall, with the highest possible score being 1 and the lowest score being 0 [43]. The calculations are as follows:

- Precision = TP / (TP + FP)
- *Recall* = *TP / (TP* + *FN)*
- *F-measure* = *2* × *precision *×* recall/(precision* + *recall)*

An ASV percent abundance threshold per mock per pipeline was set as a quality control measure for subsequent analyses to remove spurious sequences that increase FP. All quality control analyses were performed on paired-end pipelines for each reference database (GG and SILVA). To determine an appropriate threshold, we calculated overall precision, recall, and F-measure for all paired-end pipelines per mock at ASV abundance thresholds of 0%, 0.01%, 0.05%, 0.1%, 0.5%, and 1%, where 0.01% means that ASVs within a particular pipeline/mock combination that have a proportional abundance below 0.01% are excluded. Per reference database, we then calculated the precision, recall, and F-measure averages for each pipeline-threshold combination to contrast their performance. The ASV abundance threshold of 0.01% and the SILVA reference database had the best performing pipelines and highest overall F-measure means, and therefore were selected for all subsequent analyses.

## Results

Our goal was to evaluate the performance of read concatenation using different bioinformatic pipelines for the analysis of 16S rRNA gene sequences to accurately assign taxonomy. The 16S rRNA gene sequence data from four previous studies were used [35–38] and represented ten mock communities (A1, C1, G1–G4, S1–S4). All mocks were sequenced with paired-end reads on MiSeq platforms but differed in the number of samples, bacterial makeup, average read quality and read length, and primer sets covering the 16S rRNA gene variable regions: V1–V3 (A1), V3–V4 (S3–4) or V4 only (C1, G1–4, S1–2; Additional file 3: Table S1). Each pipeline generated a list of ASVs for each mock, which were then taxonomically identified to the bacterial genus level. Using the list of genera detected from each pipeline, the presence and absence of each mock community bacterial member were used to determine the number of genera that were true positives (TPs), false positives (FPs), and false negatives (FNs). These were used to calculate the precision, recall, and F-measure per pipeline per mock community (see “Methods” section). We compared the taxonomic recovery from different pipelines with regard to the influence of the reference database, sequence trimming characteristics, and single-end vs paired-end read processing and merging (Fig. 1).

### Filtering low-abundance ASVs improves accuracy

We applied a range of ASV abundance thresholds per mock per paired-end pipeline to evaluate the impact of low abundant sequences on taxonomic recovery. As the abundance threshold for filtering ASVs increases, there is generally an increase in precision (fewer FPs) but a decrease in recall (fewer TPs). For both GG and SILVA, 0.01% had the highest F-measure compared to other thresholds, striking the best balance between precision and recall (Additional file 2: Fig. S2). SILVA had a higher F-measure average than GG at every threshold. For SILVA, 0.01% had the highest F-measure (0.812), followed by 0.05% (0.804), 0.1% (0.798), 0% (0.774), 0.5% (0.728), and 1% (0.657). For GG, 0.01% also had the highest F-measure (0.731), followed by 0.05% (0.717), 0% (0.709), 0.1% (0.707), 0.5% (0.639), and 1% (0.572). Each threshold affects the total numbers of TPs, FPs, and FNs per pipeline, with higher thresholds losing more FPs at the expense of losing TPs and gaining FNs (Additional file 2: Fig. S3). Not applying an ASV abundance threshold retained more than twice as many FPs as other thresholds and therefore resulted in poor precision. A 0.01% threshold was selected for all subsequent analyses.

### Reference databases impact taxonomic recovery

**Fig. 3:**
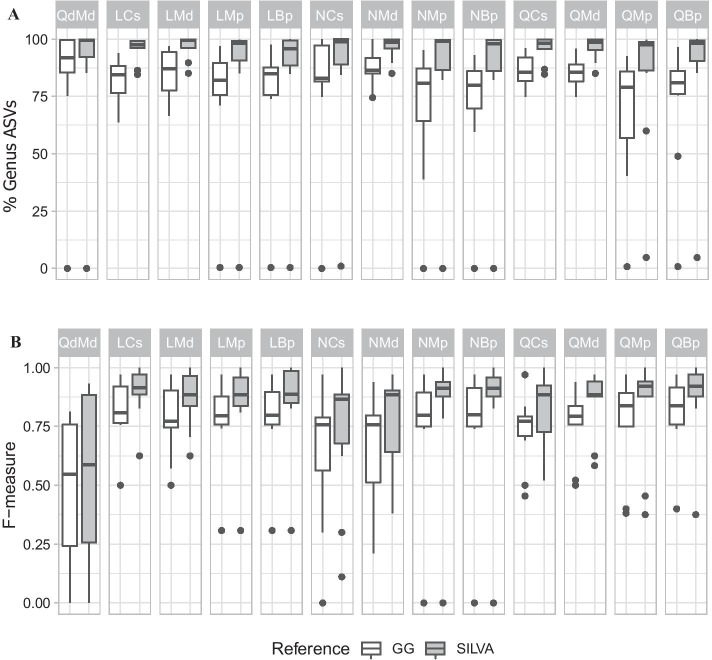
Reference database performance comparison between Greengenes (GG) and SILVA. **A** Distribution of the percent of ASVs classified to the genus level across pipelines. **B** Distribution of F-measure across pipelines. Distributions consider all ten mock communities, with medians shown inside boxplots. SILVA has more ASVs identified to the genus level than GG, including more TPs and fewer FPs, resulting in a higher average F-measure in all pipelines

The performance of the reference databases GG and SILVA was evaluated using the percent of ASVs identified to the bacterial genus level and genus level F-measures. It is important to note that all sequence processing steps are the same up until taxonomic classification using the reference database, meaning that a pipeline has the same ASVs used for taxonomic identification in both GG and SILVA. Pipelines using SILVA were consistently able to identify more ASVs to the genus level for all mock communities, with on average 10% more ASVs (mean 89.817% vs 78.475% respectively) (Fig. 3A). In addition, every pipeline had a higher mean F-measure with SILVA compared to GG (Fig. 3B). SILVA had higher precision than GG and had particularly better recall (by 9%), leading to superior mean F-measure by an average of 8% (SILVA had precision = 0.926, recall = 0.769, and F-measure = 0.812 compared to GG with precision = 0.875, recall = 0.664, and F-measure = 0.731). Some taxa were not identified with either database, despite there being reference sequences for all genera in both databases with the exception of *Howardella* and *Herpetosiphon* missing from the GG database. Overall, SILVA identified 8 genera that GG missed, GG identified 1 genus that SILVA missed, and both GG and SILVA shared 15 FPs in total, with GG having an additional 7 unique FPs and SILVA having an additional 6 (Additional file 3: Table S5). There were occasionally differences between databases of abundant taxa (> 10% abundance) that were identified as confamilial genera (e.g., Dorea vs Coprococcus in mock C1), or only classified to the family level with GG (e.g., unknown Enterobacteriaceae in mock G3, Escherichia in SILVA). Because SILVA performed better than GG overall, all further analyses to contrast pipeline performance are presented using the results from taxonomy with SILVA as the reference.

### Read trimming performance depends on read quality

**Fig. 4:**
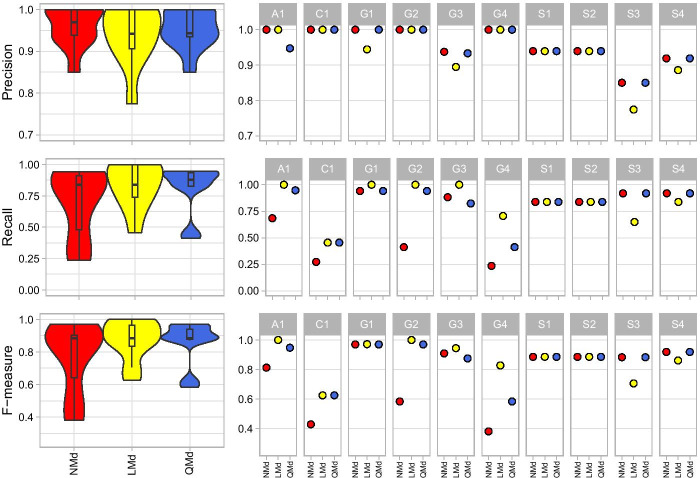
DADA2 merged pipeline comparisons showing the precision, recall, and F-measure averages across all mocks per pipeline, and all pipelines per mock

The effects of trimming were evaluated using taxonomic classifications from DADA2-merged paired-end reads using non-trimmed (NMd), length-trimmed (LMd), and quality-trimmed data (QMd and QdMd, where QdMd has the same non-trimmed input as NMd but undergoes quality trimming directly in DADA2). Because our pipelines all used the default read quality filtering in DADA2 to remove reads with maximum expected error greater than 2 (maxEE = 2), mock communities with poorer overall read quality were left with fewer reads for taxonomic assignment (in particular mocks G1-4, Additional file 3: Table S3). The quality trimming parameters used in DADA2 (QdMd) generally resulted in fewer reads after filtering including no reads passing filtering for one mock community (G2), which had the lowest overall F-measure (mean = 0.551) for pipelines that were DADA2 merged. QdMd results were therefore excluded from further comparisons. The next lowest F-measure mean was NMd (0.766), followed by QMd (0.854), and LMd (0.871) (Table 3; Fig. 4). NMd suffered from low recall in mock communities with lower read quality (A1, C1, and G1-4) indicative of a high rate of FNs due to the filtering of reads in DADA2 leaving less than 14,000 reads (5%) for classification in each mock (Additional file 3: Table S3). NMd performed better in mocks S1-4 where read quality was high throughout the read lengths, having more reads left for taxonomic classification (with more than 350,000 reads for mocks S1-2 and S4, and 77,757 for mock S3) and sometimes achieving a higher F-measure than even trimmed pipelines (e.g., 0.883 and 0.919 compared to 0.706 and 0.861 for LMd in mocks S3 and S4 respectively) (Additional file 3: Table S6). QMd also performed better than LMd in mocks S3-4 where read quality was high. LMd retained less than half the number of sequences post DADA2 in mocks S3 and S4 than both NMd and QMd resulting in fewer TPs and poorer performance, but otherwise had higher or equal F-measure when compared to NMd and QMd in all other mocks. LMd also retained more sequences than QMd for the taxonomic classifier in every mock except G2 and S3–4 (Additional file 3: Table S3). Despite losing data at the trimming step, both LMd and QMd achieved higher mean F-measure scores than NMd. The differences in performance between the length-trimmed and quality-trimmed pipelines varied by mock community (Additional file 3: Table S6), with the former retaining more sequences and having a slightly higher average F-measure.

**Fig. 5:**
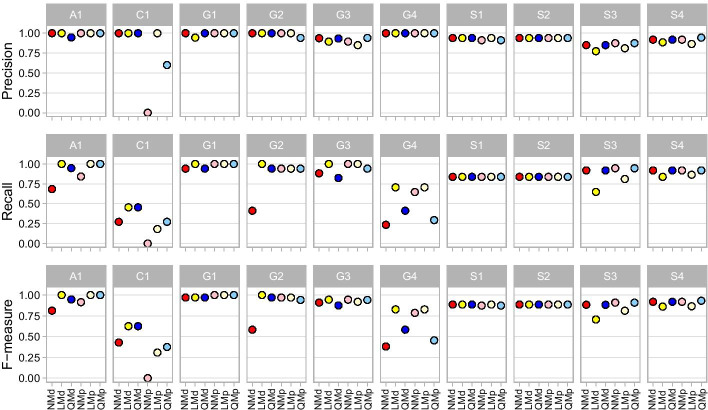
Performance comparison among DADA2 merged pipelines NMd, LMd and QMd and PANDAseq merged pipelines NMp, LMp and QMp

Because DADA2 was designed to process paired-end reads and perform its own read merging, it was unknown how already-merged (or concatenated) reads would perform in QIIME 2. Therefore, we tested if sequences merged in PANDAseq (NMp, LMp, and QMp) performed similarly in taxonomic recovery to the sequences merged in DADA2 (NMd, LMd, and QMd), before comparing the effects of concatenating vs merging with PANDAseq. The average F-measure score for NMp (0.820) was higher than NMd (0.766), whereas the trimmed PANDAseq pipelines had lower F-measure (LMp = 0.847 and QMp = 0.831) compared to those trimmed in DADA2 (LMd = 0.871 and QMd = 0.854) (Table 3), although this varied by mock community (Fig. 5; Additional file 3: Table S6). The major difference was due to the poor performance of PANDAseq pipelines in mock C1, where they had particularly higher FPs and FNs. Although the three PANDAseq merged pipelines retained a higher number of sequences for taxonomic classification in every mock community than the DADA2 merged pipelines (Additional file 3: Table S3), the majority of reads in C1 were unable to be identified to the genus level. After excluding C1, the PANDAseq pipelines had higher average F-measure scores than the DADA2 pipelines (NMp = 0.911 > NMd = 0.803; LMp = 0.907 > LMd = 0.898; QMp = 0.882 > QMd = 0.880). As the standard merged PANDAseq pipelines performed comparably well to the DADA2 merged pipelines, we proceeded to analyze the performance of concatenating paired-read either in addition to merged reads (Bp) or on their own (Cs).

### Concatenating paired-end reads can improve taxonomic recovery

**Fig. 6:**
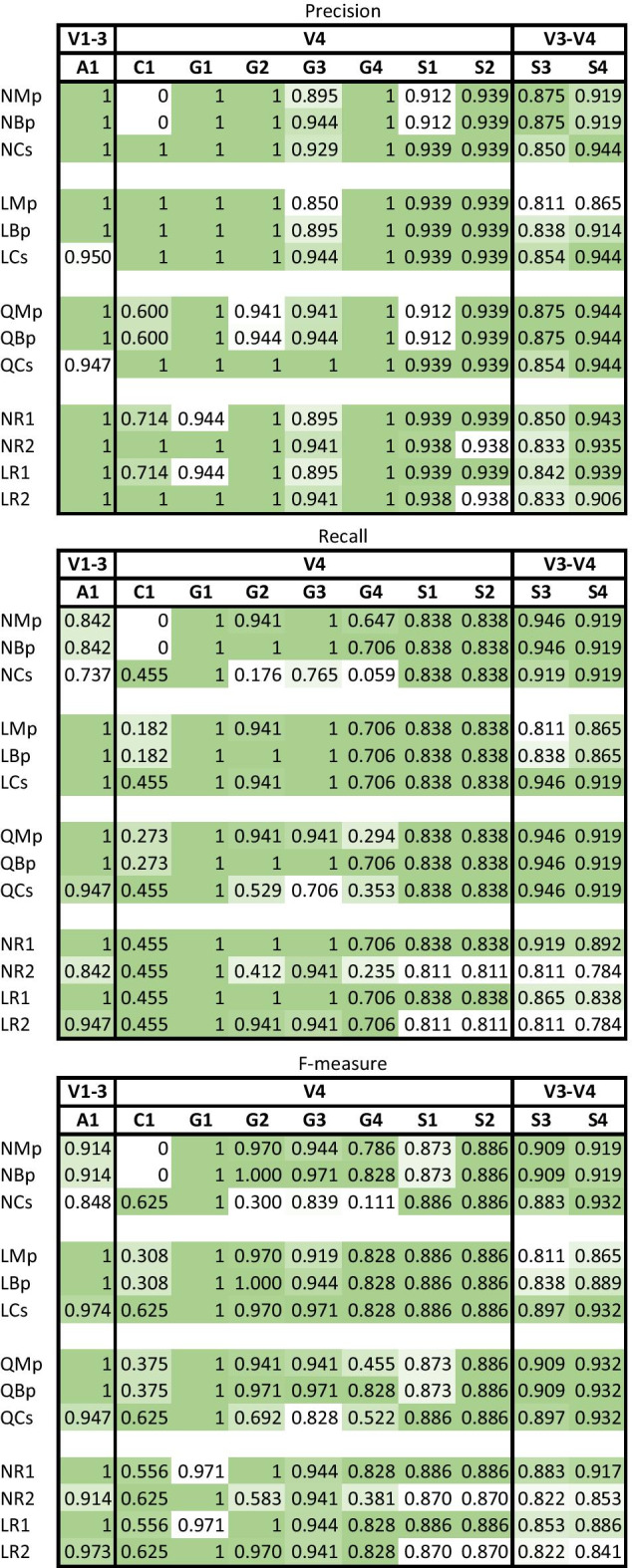
Pipeline precision, recall, and F-measure performance per 16S gene region. Color gradient is applied to each column where darker green signifies a pipeline having the highest metric for that mock community, and white signifies the lowest metric for that mock community

Trimming reads can result in excluding sequences that have insufficient overlap for merging between paired reads. To determine the impact of losing these unmerged reads, we implemented pipelines that used the tool PANDAseq to merge R1 and R2 reads in addition to adding reads unable to merge as concatenated sequences to the same file (Bp), an option currently not available with DADA2. The addition of concatenated sequences that would have otherwise been discarded in the merging process have the potential to provide better taxonomic recovery than merged reads alone simply by keeping more sequences for taxonomy. This could also apply to fully concatenated (Cs) reads as no reads are lost compared to merging non-overlapping reads, provided that concatenated sequences are able to be processed by DADA2 and do not hinder taxonomic assignment. Since non-trimmed pipelines performed poorly overall with the lowest average F-measures (Table 3; Fig. 6), we focus on length-trimmed and quality-trimmed pipelines for this analysis.

**Fig. 7:**
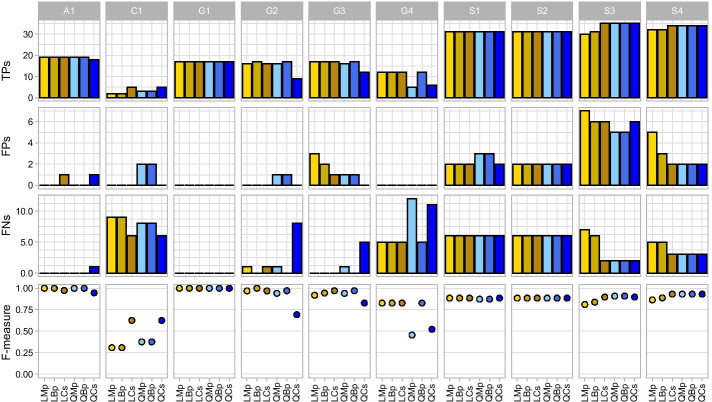
Distribution of true positives (TPs), false positives (FPs), false negatives (FNs), and F-measure per mock for length trimmed (LT) and quality trimmed (QT) pipelines combined with merged (M), merged and concatenated (MC), or solely concatenated (C) reads

Compared to merged pipelines (LMp and QMp), merged and concatenated pipelines (LBp and QBp) always resulted in keeping more sequences for taxonomy (Additional file 3: Table S3). However, even though fully concatenated pipelines (LCs and QCs) started out with the most sequences, the number was dramatically reduced by DADA2 that removes reads below a quality threshold (maxEE = 2), often reducing the percentage of input reads by more than half in mocks C1, G1–4, and A1 (mean: LMp = 67.70% > LCs = 28.593%, QMp = 80.717% > QCs = 22.175%). There was less of a difference in mocks S1-4, which have the highest average base quality of all mocks (Table 1), though concatenated pipelines still output lower percentages of input reads (mean: LMp = 96.182% > LCs = 92.975%, QMp = 95.938% > QCs = 89.368%). Despite this loss of data generally resulting in fewer ASVs for concatenated pipelines compared to merged and merged-concatenated pipelines, in most cases this did not diminish TPs (Fig. 7).

Averaging across all mocks, LCs had the highest overall F-measure (0.897), followed by QBp (0.874), LBp (0.858), LMp (0.847), QMp (0.831), and QCs (0.821). Whereas trimming had an impact on the performance of concatenated sequences in the absence of merged reads (LCs vs QCs), the addition of concatenated sequences to merged reads (Bp) resulted in improved taxonomic recovery compared to merged reads alone (Mp) for both length-trimmed and quality-trimmed pipelines: LBp and QBp had the same or better F-measure than LMp and QMp respectively in every mock (Additional file 3: Table S6). However, the top performing pipeline was not the same for every mock community. The mocks with the highest average sequence quality (S1–S4 with Q =  > 35 in both paired reads; Table 1) tended to show better performance in quality-trimmed vs length-trimmed pipelines (Fig. 7). The opposite tended to be true for the other mocks, particularly for mocks with lower read qualities (G2, G3, and G4), which had especially poor F-measures for QCs. The two pipelines with consistently high F-measures per 16S rRNA gene variable regions (either the highest or second highest per mock) were LCs and QBp, and both had higher F-measure means than all DADA2 merged pipelines (Table 3). The next best performing pipeline overall was LMd, which used DADA2 merged reads without concatenation, but this pipeline performed poorly in mock communities with large amplicons (S3 and S4), suffering from low precision and low recall.

### Pipeline performance varied by amplicon and by mock community

The target amplicon length influenced pipeline performance (Fig. 6). In general, length trimming outperformed quality trimming of V4 mocks as indicated by higher F-measures. However, quality trimming outperformed length trimming of the V3–4 mocks. Length trimming and quality trimming gave similar results for the V1–3 mock A1, where read merging outperformed pipelines containing just concatenated reads regardless of length or quality trimming. Conversely, fully concatenated pipelines had the highest F-measure in V4 mocks containing closely related bacteria (C1). The pipeline with the highest average F-measure (LCs) had the highest F-measure in seven of the ten mock communities (C1, G1, G3, G4, S1, S2, S4) all but one of which were V4 mocks. QBp, the pipeline with the second highest average F-measure, also had the highest F-measure in seven mock communities including the V1–3 mock (A1), both V3–4 mocks (S3–S4), and V4 mocks (G1, G3, G4, and S2).

The amplified 16S rRNA gene region V1–3 (mock A1) was best represented by LMp, LBp, QMp, and QBp with an F-measure of 1, identifying all 19 mock bacteria (Additional file 3: Table S7). LCs captured all these genera but had an additional FP (F-measure = 0.974), while QCs had 1 FN and 1 FP (F-measure = 0.947). In contrast, LCs had the highest F-measure in every mock with the V4 16S rRNA gene region except G2 (QBp = 0.971 > LCs = 0.970). In mock C1, which is made up of closely related bacteria, fully concatenated pipelines LCs and QCs had the highest F-measures (0.625) with just 5 out of 11 TPs but 0 FPs (Additional file 3: Table S7), 2 more TPs than the next best pipelines. In mock G1, all the different pipelines had an identical F-measure of 1, meaning all 17 genera were identified with no FPs. G2, G3, and G4 mocks were better represented with length-trimmed pipelines than quality-trimmed. The exception here was QBp that tied for the highest F-measure scores in mocks G3 and G4, and performed worse than only LBp in G2 (Fig. 6). In mock S1, pipelines LMp, LBp and LCs performed equally well (F-measure = 0.886). QCs also had an F-measure of 0.886, outperforming QMp and QBp (0.873) in mock S1. All six of these pipelines had an F-measure of 0.886 for mock S2. The V3–4 region (mocks S3–4) was best represented by QMp and QBp. Both had F-measures of 0.909 for mock S3 and 0.932 for mock S4. QCs and LCs performed just as well in mock S4 but slightly worse in S3.

### Balanced mock communities had better taxonomic recovery than unbalanced mocks

Differences were detected among pipelines between three V4 balanced communities and their matching unbalanced communities (where taxa abundances varied within the mock community): G1 vs G3, G2 vs G4, and S1 vs S2 respectively (Additional file 2: Fig. S4). As expected, balanced communities had a more even spread of relative abundance proportions per taxa, which was consistently observed across pipelines (mean Pielou’s evenness J for G1, G2 and S1 = 0.89, 0.86, 0.89 respectively, compared to G3, G4, and S2 = 0.57, 0.61, 0.83, respectively). Balanced communities also had higher alpha diversity than unbalanced communities (mean Shannon’s diversity H for G1, G2 and S1 = 2.57, 2.21, 3.12 compared to G3, G4, and S2 = 1.63, 1.27, 2.94). This higher diversity in balanced communities was mainly due to more TPs (mean TPs for G1, G2 and S1 = 16.0, 13.3 and 30.8 compared to G3, G4, and S2 = 15.1, 8.5 and 30.8) than FPs (mean FPs for G1, G2 and S1 = 1.7, 1.6 and 3.3 compared to G3, G4, and S2 = 2.7, 1.5 and 3.0). Among LMp, LBp, LCs, QMp, QBp, and QCs, the balanced mocks had higher F-measure scores in every instance except QMp and QBp in which S2 outperformed S1. Single-end pipelines performed worse in the unbalanced communities as well.

### Single read analysis is rarely better than concatenating paired reads

**Fig. 8:**
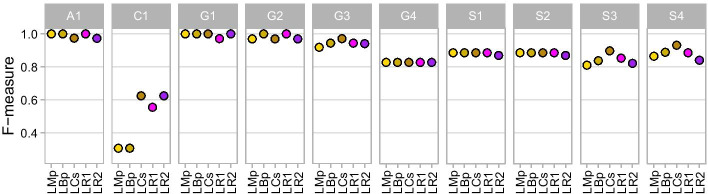
F-measure of length-trimmed single-end and paired-end pipelines per mock community

We evaluated the performance of pipelines using single-end reads compared with paired-end analyses following read merging and/or concatenation. We were particularly interested in knowing whether concatenation could replace single-end analysis when reads do not overlap, therefore keeping longer sequences for increased taxonomic resolution. We focused our analysis on length-trimmed and non-trimmed single-end processing because length-trimmed paired-end pipelines on average outperformed their quality-trimmed counterparts (Table 3). Similar to paired-end pipelines, single-end pipelines that used length-trimmed reads outperformed non-trimmed reads, with the same or higher F-measure scores in all mocks except in S3 (NR1 = 0.883 > LR1 = 0.853) and S4 (NR1 = 0.917 > LR1 = 0.886; NR2 = 0.853 > LR2 = 0.840; Fig. 6; Additional file 3: Table S6). We therefore present the performance of length-trimmed single reads (LR1 and LR2) with length-trimmed paired-end pipelines (LMp, LBp, and LCs) across mock communities. Read concatenation usually yielded greater F-measures than single reads: the paired-end pipelines LBp and LCs each outperform or are equal to LR1 in 8 of 10 mocks and to LR2 in 9 (LBp) or all 10 (LCs) mocks (Fig. 8; Additional file 3: Table S6). LR2 had lower overall recall, particularly in the S1–4 mocks (Fig. 6).

The number of reads retained for taxonomic classification in single-end vs paired-end pipelines seldom correlated with F-measure performance. For example, the pipelines with the highest F-measure in mock C1 (LCs and LR2) had the lowest number of reads post-filtering for taxonomic classification (LCs: 863,065 and LR2: 1,064,603 compared to LMp: 1,853,002, LBp: 1,853,336 and LR1: 1,455,823; Additional file 3: Table S3). In mocks G2 and G3, LR2 had the highest number of sequences for taxonomic classifications (88,998 and 267,098) but had lower F-measure than LR1, which had less than half the number of sequences (16,358 and 53,303). LCs had the same F-measure as LR2 in G2 with only 35,775 sequences and the best F-measure in G3 with 96,227 sequences. For mocks S1–2 targeting the V4 region, all pipelines retained over 90% of input reads and except for LR2 had an F-measure of 0.886. For mocks S3–4 targeting the V3–4 region, all pipelines also retained over 90% of input reads, but there was greater variance in performance with greater numbers of FPs (between 2–7) than for mocks S1–2 that comprised the same taxa. LCs had the highest F-measure in mocks S3–4, outperforming both single-end pipelines (Fig. 8; Additional file 3: Table S6).

## Discussion

The loss of data in microbial amplicon sequencing analysis resulting from the inability to merge paired reads can hinder species recovery and limit the appropriateness of comparing taxonomic profiling results across studies. Successful read pair merging depends on numerous technical and biological factors including amplicon and read lengths, sequence quality and trimming, and biological sequence (length) variation among target community members. Here we use mock communities that vary in these factors to evaluate the accuracy of taxonomic recovery among bioinformatic pipelines implemented in QIIME 2 that differed in abundance filtering thresholds, reference databases, read trimming, and in particular the use of read concatenation instead of or in addition to read merging. The performance of individual pipelines varied by mock community, but we found that read concatenation was usually advantageous in detecting true positives while limiting false positives.

### Abundance filtering influences type I and type II errors

Filtering low abundant ASVs or OTUs is typically carried out to reduce the impact of spurious sequences and improve diversity estimates. In our study, an ASV abundance threshold of 0.01% gave on average the best overall balance between precision and recall. Compared to not filtering by abundance, this threshold reduced the number of FPs while retaining the majority of TPs. We found an ASV abundance threshold of 0.5% and 1% completely eliminated FPs in all conditions but with a significant loss of TPs. There is substantial variation in the abundance thresholds applied across other microbial studies, for example 0.1% [5], 0.01% [36], 0.005% [3] and 0.0005% [45]. The exact effects of different thresholds on TPs and FPs will depend on the particular sequence library characteristics such as the sequencing depth and microbial community diversity as well as the bioinformatic pipeline, but our results show consistent trends regarding average precision and recall across pipelines used in this study.

### Reference databases affect taxonomic recovery

Two of the most commonly used reference databases for taxonomic identification of 16S rRNA gene sequences are Greengenes (GG) and SILVA [5, 31]. Despite both databases containing reference sequences for each genus in the mock communities used in this study (with the exception of two genera in GG), we found differences in the TPs or FPs in every mock community between GG and SILVA, sometimes even using the same bioinformatics pipeline. This is likely an influence of reference database content on taxonomic resolution – depending on how many closely related sequences a database has to the focal sequence, it can create more uncertainty in identifying the correct taxon [43]. It is also possible that the training of the taxonomic classifier in QIIME 2 influenced the performance of the different databases.

SILVA performed better than GG in all mocks used in this study. GG has not been updated since 2013, is no longer maintained, and has the smallest taxonomic classification and thus least diversity out of the major reference databases [31]. Regardless, GG is still commonly used [19, 26, 36, 43, 45, 46], and it has been shown to have higher accuracy than the Ribosomal Database Project (RDP) for characterizing some bacterial communities [3]. Its continued use also has practical reasons; researchers can compare their taxonomic results to a previous study that used GG, or they might already have an in-house alignment process setup that uses this database. SILVA, on the other hand, is actively maintained and releases multiple updated versions every year, making it a popular choice for sequence identification. Additionally, SILVA shares more taxonomic units with the comprehensive NCBI taxonomy database than both GG and RDP [31]. A recent study comparing microbiomes using two different sequence processing tools and taxonomic classification with GG and SILVA found no differences between any tool-database combination for those taxa abundant at more than 10%, but substantial differences in reported taxonomy across tools for bacteria with abundance levels under 10% with GG [5]. In our study, taxonomic discrepancies between GG and SILVA classifications in multiple pipelines were even found in the bacteria abundant at more than 10%, demonstrating potentially strong influences of reference databases on taxonomic characterizations depending on the microbial community composition.

### Read quality affects pipeline performance rankings

Trimming reads before sequence analysis is important to remove sequencing primers and poor-quality bases that, when erroneous, can interfere with accurate taxonomic assignment [30]. However, trimming can itself hinder taxonomic resolution if it removes regions of the amplicon necessary to distinguish among closely related organisms, or if paired reads become too short for merging and are discarded entirely. In our study, non-trimmed pipelines indeed usually retained more sequences for taxonomic classification, but only in mocks with high average read quality. For most mocks, not trimming reads was counterproductive because it increased the number of low-quality bases in reads that led to more read filtering at later steps; DADA2 uses a maximum expected error (maxEE) of 2 by default, which removes entire reads that have more than 2 expected errors throughout the read before taxonomic classification. Future studies might consider relaxing the maxEE filter in DADA2 to retain more sequences and potentially improve taxonomic recovery. Although non-trimmed pipelines sometimes outperformed trimmed pipelines, there was always at least one length-trimmed or quality-trimmed pipeline combined with read concatenation that performed just as well or better than non-trimmed pipelines (Fig. 6). This indicates that, although trimming may not always be required for optimal taxonomic recovery, including concatenated trimmed reads helped overcome the shorter reads from trimming.

### Concatenating paired reads increases performance over merged and single reads

Trimming raw FASTQ sequences by length at user-specified positions can be advantageous over quality trimming for OTU clustering [17], as well as for reducing the number of ASVs that would come from trimming the same variant to different lengths. We found the average F-measure of merged length-trimmed (LMp) to be higher than quality-trimmed (QMp), even though the majority of quality-trimmed pipelines had more merged sequences than their length-trimmed counterparts. When adding concatenated reads to merged reads, this always resulted in more sequences for taxonomic classification and the same, if not better, F-measure scores than just merged pipelines. Quality trimming (QBp) retained more sequences for taxonomy in most mocks and resulted in a higher mean F-measure than length trimming (LBp). QBp had the second highest average F-measure of all pipelines including QMp and LMp, indicating that the loss of sequence information during merging was largely recovered with the addition of concatenated sequences. On the other hand, fully concatenated pipelines like QCs had fewer sequences left for taxonomy following DADA2 compared to pipelines with merged reads (QMp and QBp) in most mock communities, despite starting with more ASVs. Similar to non-trimmed reads, the act of concatenating reads instead of merging them at their ends increases the number of bases in the sequence, particularly those that have lower Phred scores (at the 3′ end of reads). Concatenated reads therefore are more prone to filtering by the default maxEE threshold in DADA2 unless average base quality is high throughout the read. The large number of sequences filtered diminished the performance of QCs. Conversely, length-trimmed concatenation (LCs) outperformed all other pipelines with the highest overall mean F-measure, despite undergoing high levels of sequence filtering before taxonomy. An important caveat is that the taxonomic classifier needs to accommodate for concatenated sequences that do not represent the exact amplicon sequence, with either missing sequence between the joined read ends or duplicated sequence where read pairs overlap that would otherwise have been merged. Our results show that the performance of different combinations of trimming and concatenation are affected by overall sequencing quality, and that read retention does not necessarily lead to better taxonomic resolution, at least with microbial communities of relatively low diversity as used in this study.

That the fully concatenated pipeline LCs had the highest mean F-measure shows that concatenating even overlapping paired-end reads can provide taxonomically useful information that would otherwise be lost when only considering merged or single-end sequences. In studies using long amplicons where paired-end sequences do not overlap, R2 reads are often discarded and just the R1 reads are used for analysis, even though concatenating these sequences is advantageous as long as the taxonomic classifier can handle these artificially contiguous sequences: IM-TORNADO [23], *hybrid-denovo* [18], JTax [17], and MeFiT [47] are tools able to process non-overlapping paired-end reads that concatenate R1 and R2 sequences, increasing taxonomic accuracy over R1 or R2 alone. Our findings support these earlier results, in addition to showing that concatenation often outperforms merged reads based on taxonomic classification of ASVs in QIIME 2. Cascabel [48] is a comprehensive tool allowing the user to select between either OTU or ASV pipelines and that also allows sequences to be concatenated instead of merged, but the accuracy of concatenation in this tool has not been validated. Here, we demonstrate the advantage of read concatenation over merged reads alone, R1 alone, and R2 alone for the analysis of ASVs. Because DADA2 performs denoising on the R1 and R2 reads independently before merging, paired-end reads are normally merged within DADA2 [35], but our results suggest that merging and concatenating reads prior to processing reads in DADA2 within QIIME 2 outperforms DADA2-merging under various scenarios. We expect that future tools will include read concatenation in addition to read merging to fully make use of paired-end reads, in particular when sequencing long amplicons.

## Conclusion

Correct taxonomic assignments and recovery in microbial amplicon sequence analyses are important to generate accurate estimates of diversity and community composition. Identification of taxa can be influenced by the parameters used for sequence processing and alignment, areas where consistency is lacking among researchers. This presents significant challenges in comparing results across studies. We tested the performance of sequence processing pipelines to determine, among other things, whether read concatenation instead of or in addition to read merging could improve taxonomic recovery of ASVs using a diverse set of publicly available 16S rRNA gene sequence data from mock communities. Our investigation shows that a proper minimum abundance threshold per taxa improves F-measure scores by removing very low abundant taxa (contaminants, noise, ASVs aligned with low confidence values), that an up-to-date SILVA reference database is superior to the Greengenes database, and that concatenation of sequences improves taxonomic recovery. Additionally, length trimming combined with concatenating sequences outperforms sequence merging even in 16 s rRNA gene variable regions that have a high degree of overlap between the forward and reverse reads (e.g.,V4), whereas quality trimming performs well with longer amplicons (e.g., V1-3 and V3-4). However, when sequence quality is low, length trimming preserves more sequences for taxonomic identification and thus remains an appropriate option even for longer amplicon. We have shown for the first time that inclusion of concatenated paired-end reads consistently improves accurate taxonomic assignment of ASVs across a variety of samples that differed in sequence quality, amplicon region, and read length, suggesting robust performance under various contexts of gene marker-based studies.

## Supplementary Information

**Additional file 1. Figure S1**. Phred quality plots of raw sequences for each mock community.

**Additional file 2. Figure S2**. Precision, recall, and F-measure statistic comparisons averaged across mocks for each paired-end pipeline at different ASV percent abundance thresholds. Pipelines with taxonomy performed using SILVA had a higher F-measure mean at every threshold compared to Greengenes. **Figure S3**. ASV percent abundance threshold comparison of true positives (TPs), false positives (FPs), and false negatives (FNs) among paired-end pipelines using the SILVA references database. The unique number of genera identified for each of the ten mock communities per pipelines are summer together per pipeline per threshold. Genus counts do not consider ASV abundances. **Figure S4**. Heatmaps comparing balanced (G1, G2, S1) versus unbalanced (G3, G4, S2) mock communities with the same genera compositions. A) G1 vs G3, B) G2 vs G4, and C) S1 vs S2. Color scale shows proportional abundance of taxa within each mock / pipeline. Taxa are categorized by False Positives (blue) and True Positives (red). Relative abundance scale goes from dark blue (low) to red (high).

**Additional file 3. Table S1**. Mock community sequence information and trimmed length positions. Primer information includes the name of each primer in the literature and the associated sequence, read lengths of paired reads, length of the reads after length-trimming by median Q20 score, SRA and Bioproject accessions, and citation. **Table S2**. Bacteria present within each mock community. **Table S3**. DADA2 statistics for each pipeline per mock community. Input represents the number of reads used after the trimming and merging/concatenating steps; Filtered is the amount of sequences remaining after DADA2 filtering based on a maxee=2; Denoised is the number of sequences remaining after DADA2 denoising; Merged and % of input merged applies only to pipelines with merging by DADA2 (NMd, LMd , QMd and QdMd); Non-chimeric output sequences were passed into the taxonomic classifier; % output is the percent of sequences from the input left for taxonomic classification. **Table S4**. Genera that were originally determined as false positives (FP) but have synonyms of taxa from mock communities, and therefore were renamed to match taxa from mock communities (modified names now represent true positives – TPs). **Table S5**. True positive (TP), false positive (FP), and false negative (FN) genera that are shared and unique between GG and SILVA reference databases. Mocks are grouped per study. **Table S6**. TP, FP, and FN counts for each pipeline per mock along with the calculated precision, recall, and F-measure. SILVA reference database and an ASV abundance threshold of 0.01% was used to filter out low abundant ASVs before these calculations were performed. Pipelines arranged by descending F-measure score per mock. **Table S7.** True positive (TP), false positive (FP), and false negative (FN) bacterial genera found in each mock per pipeline with SILVA database.

## Data Availability

Information associated with the mock community sequence data used in this manuscript can be found in Additional file 3: Table S1 and S2. All code used for processing the 16S rRNA gene sequences to exporting taxonomic results for analysis in R are available at https://github.com/BEEGlab/16SProcessingPipelinesWithQIIME2. The QIIME 2 full-length 16S rRNA gene Naive Bayes trained classifiers used were SILVA 132 99% OTUs full-length sequences [41] and GG 13_8 99% OTUs full-length sequences [42].
