# Supplementary material for: Concatenation of paired-end reads improves taxonomic classification of amplicons for profiling microbial communities
Source: BMC Bioinformatics. 2021 Oct 12;22:493. doi: 10.1186/s12859-021-04410-2 (PMC8507205; doi:10.1186/s12859-021-04410-2)

A1

Click and drag on plot to zoom in. Double click to zoom back out to full size. Hover over a box to see the parametric seven-number summary of the quality scores at the corresponding position.

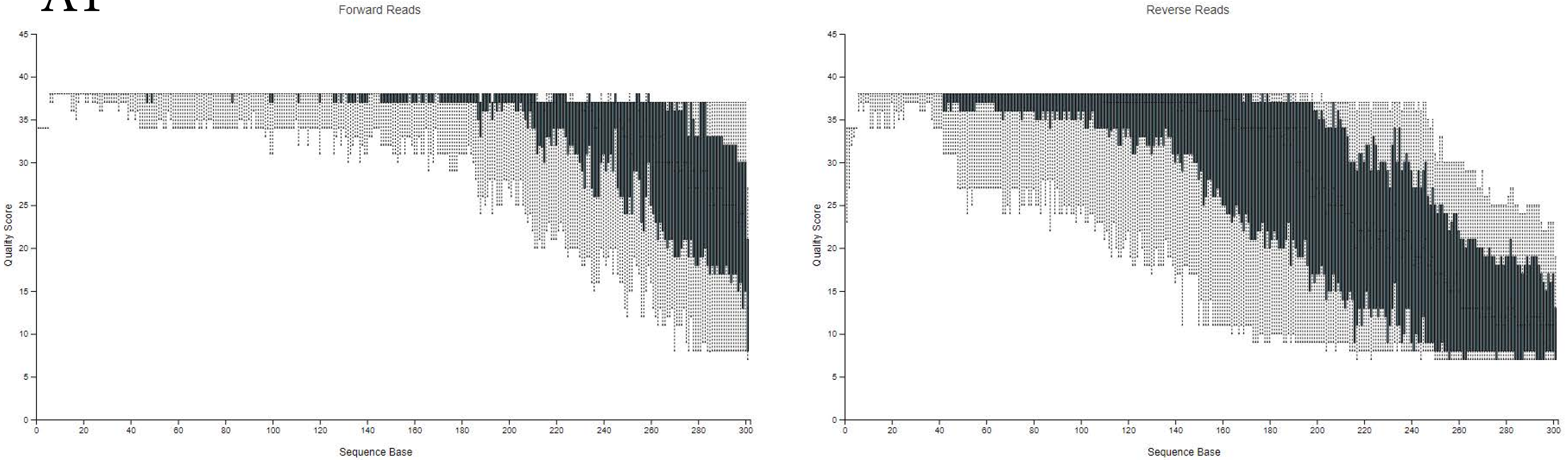

C1

Click and drag on plot to zoom in. Double click to zoom back out to full size. Hover over a box to see the parametric seven-number summary of the quality scores at the corresponding position.

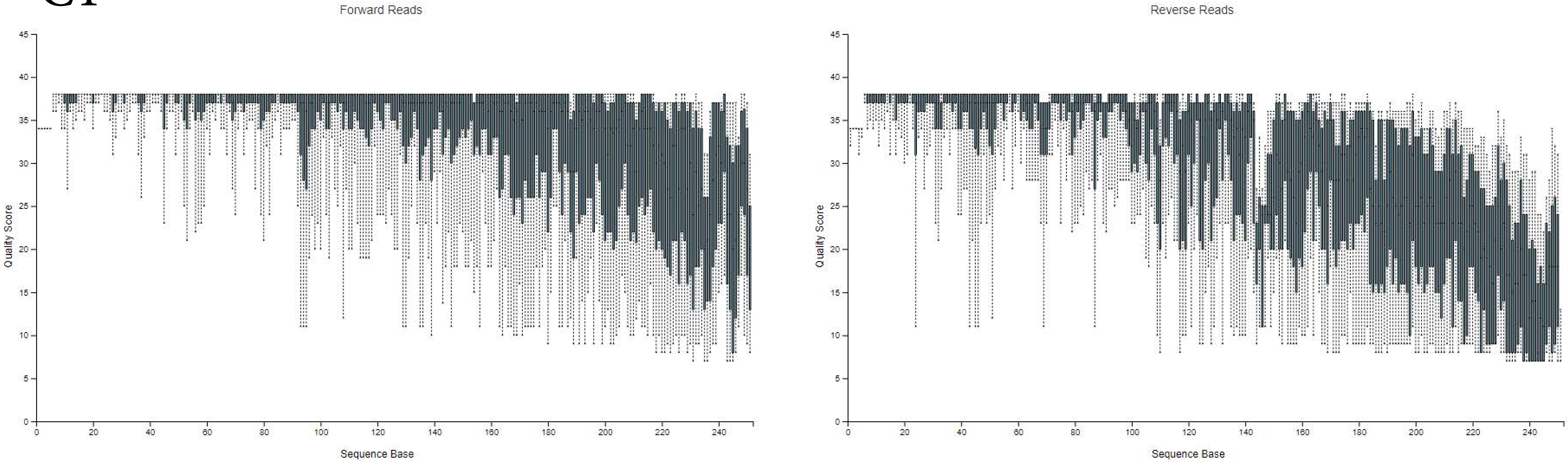

G1

Click and drag on plot to zoom in. Double click to zoom back out to full size. Hover over a box to see the parametric seven-number summary of the quality scores at the corresponding position.

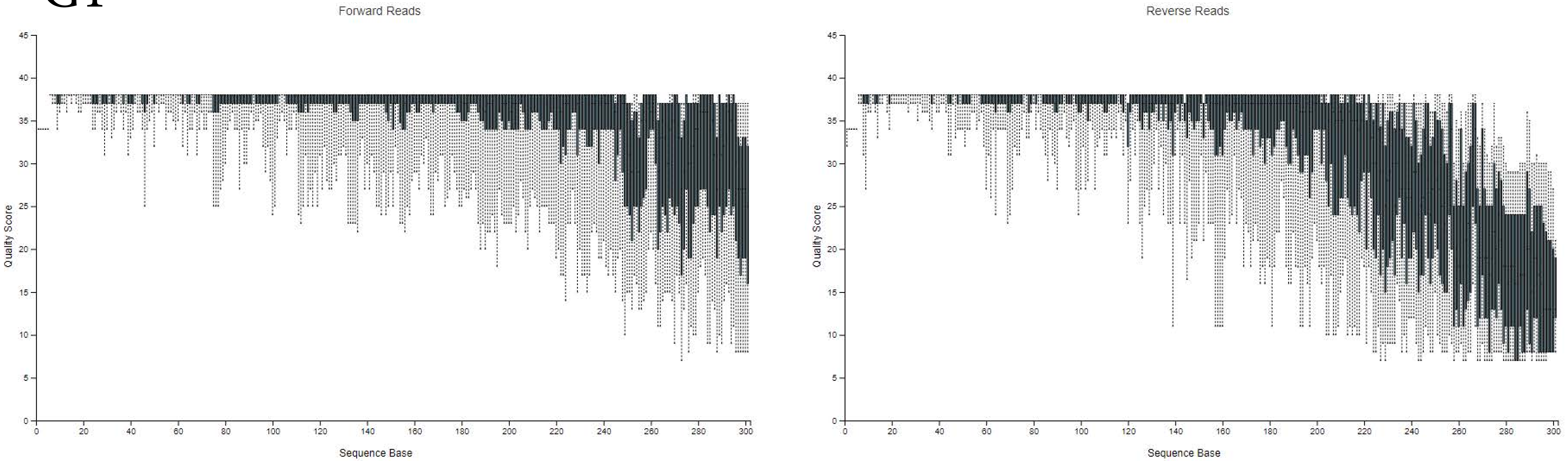

G2

Click and drag on plot to zoom in. Double click to zoom back out to full size. Hover over a box to see the parametric seven-number summary of the quality scores at the corresponding position.

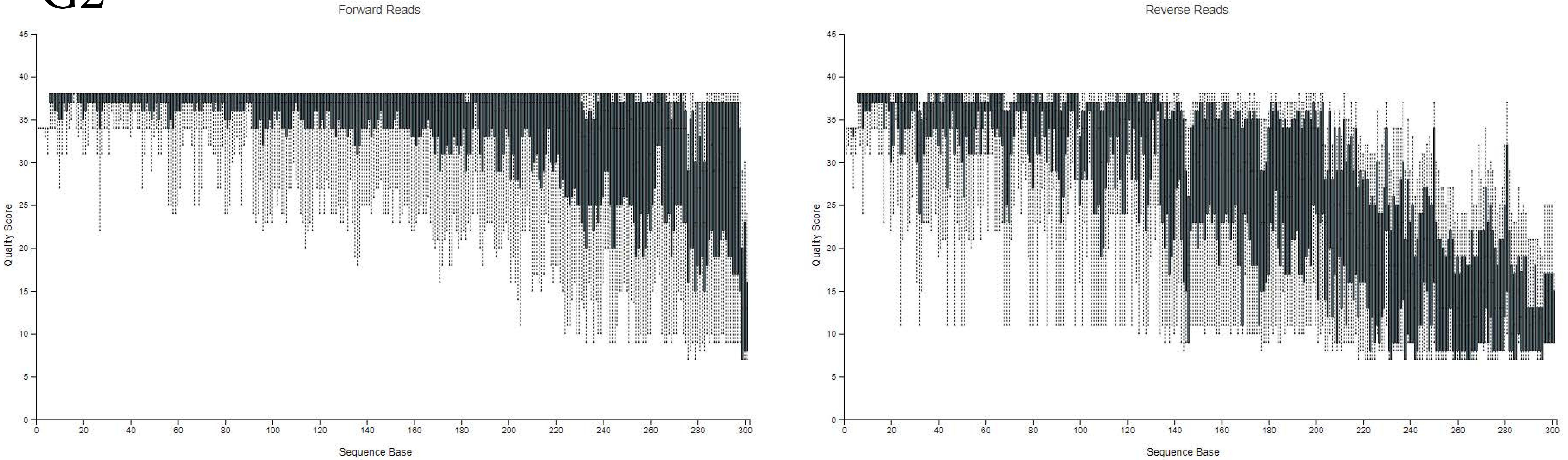

G3

Click and drag on plot to zoom in. Double click to zoom back out to full size. Hover over a box to see the parametric seven-number summary of the quality scores at the corresponding position.

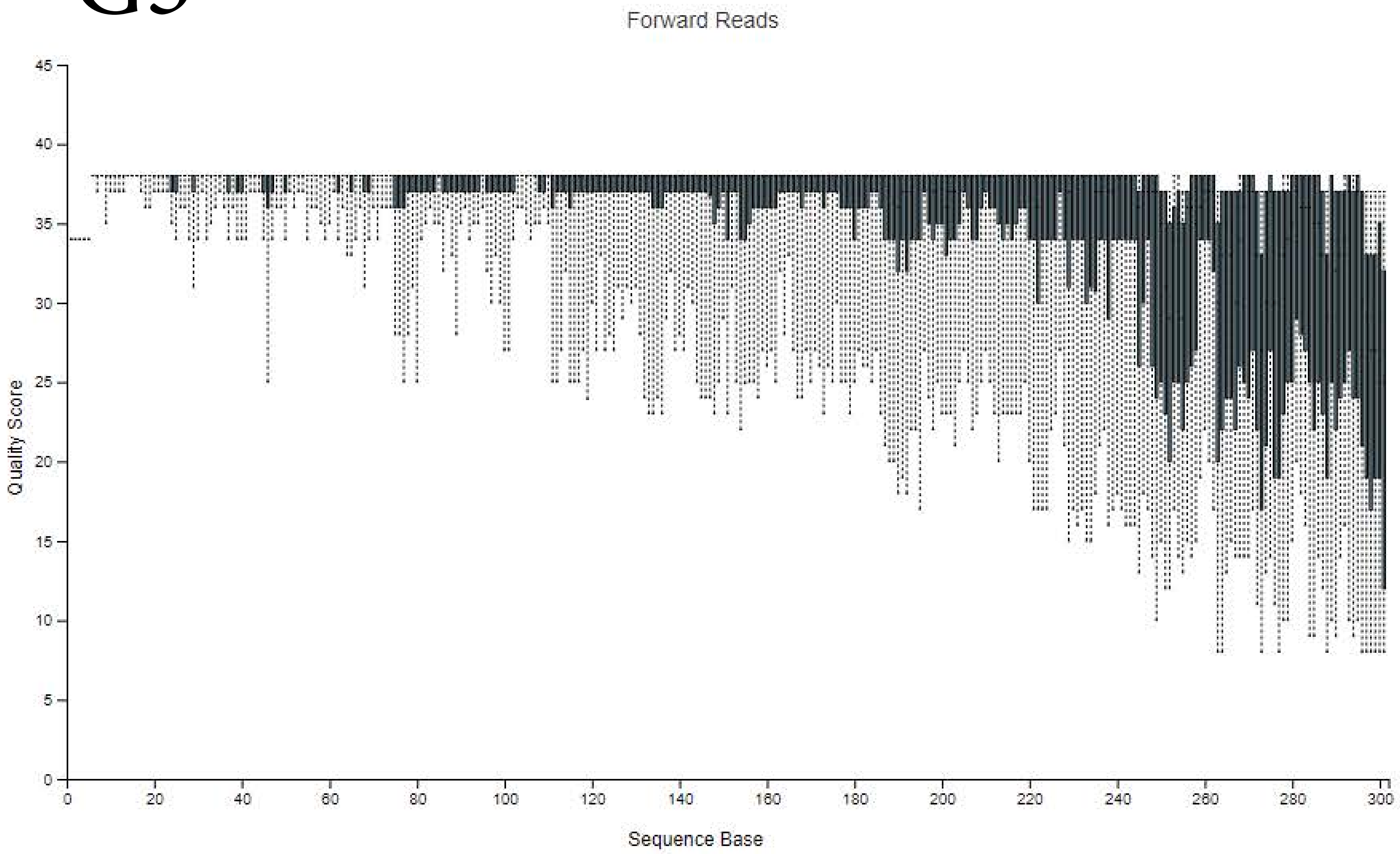

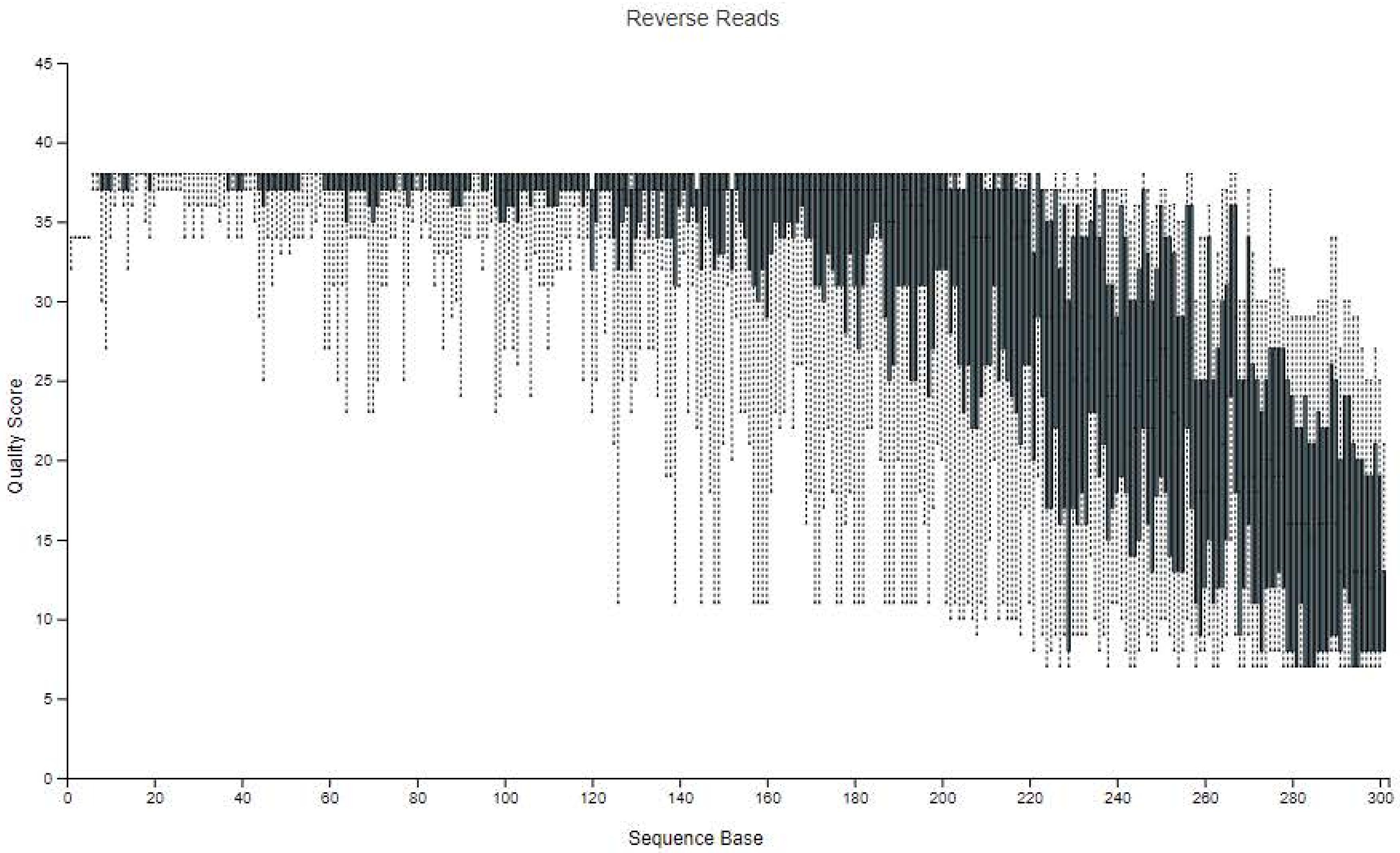

G4

Click and drag on plot to zoom in. Double click to zoom back out to full size. Hover over a box to see the parametric seven-number summary of the quality scores at the corresponding position.

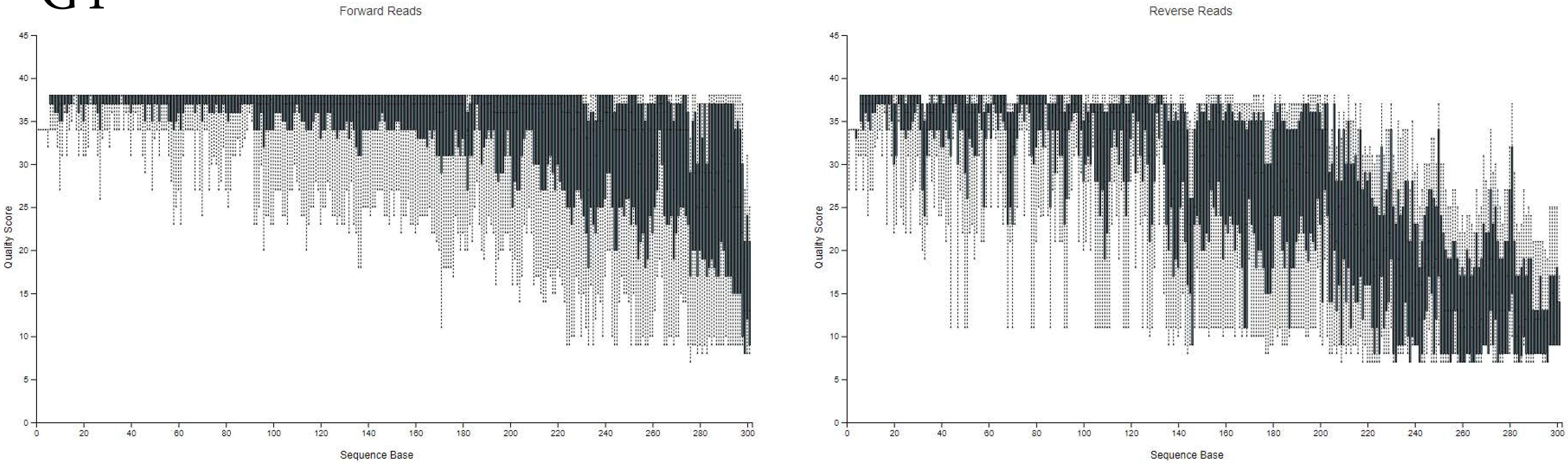

S1

Click and drag on plot to zoom in. Double click to zoom back out to full size. Hover over a box to see the parametric seven-number summary of the quality scores at the corresponding position.

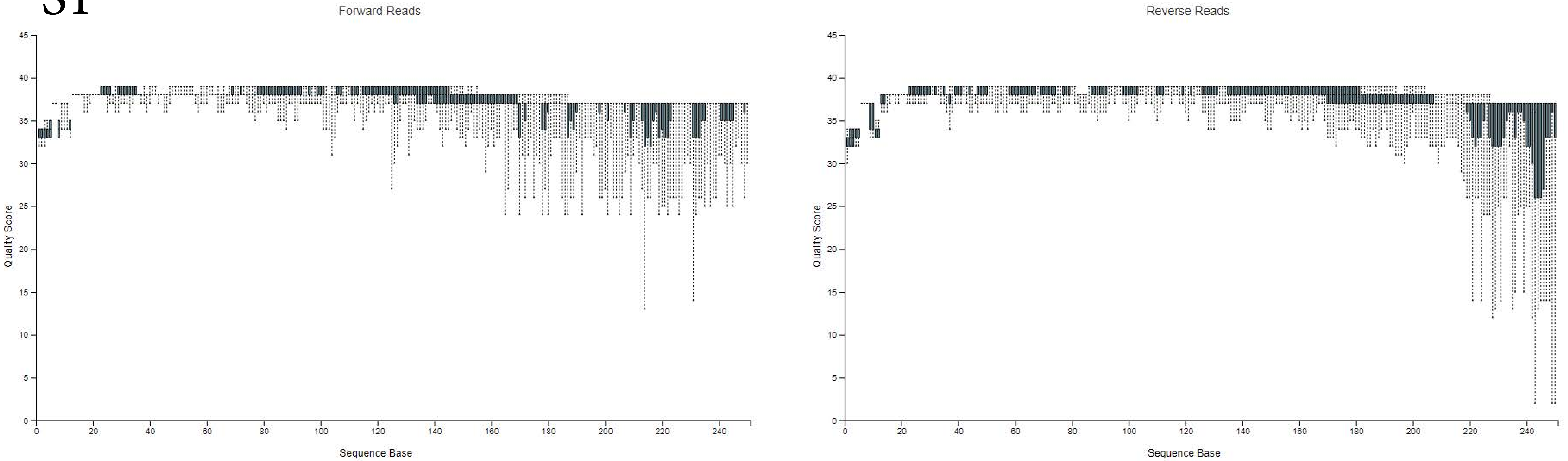

S2

Click and drag on plot to zoom in. Double click to zoom back out to full size. Hover over a box to see the parametric seven-number summary of the quality scores at the corresponding position.

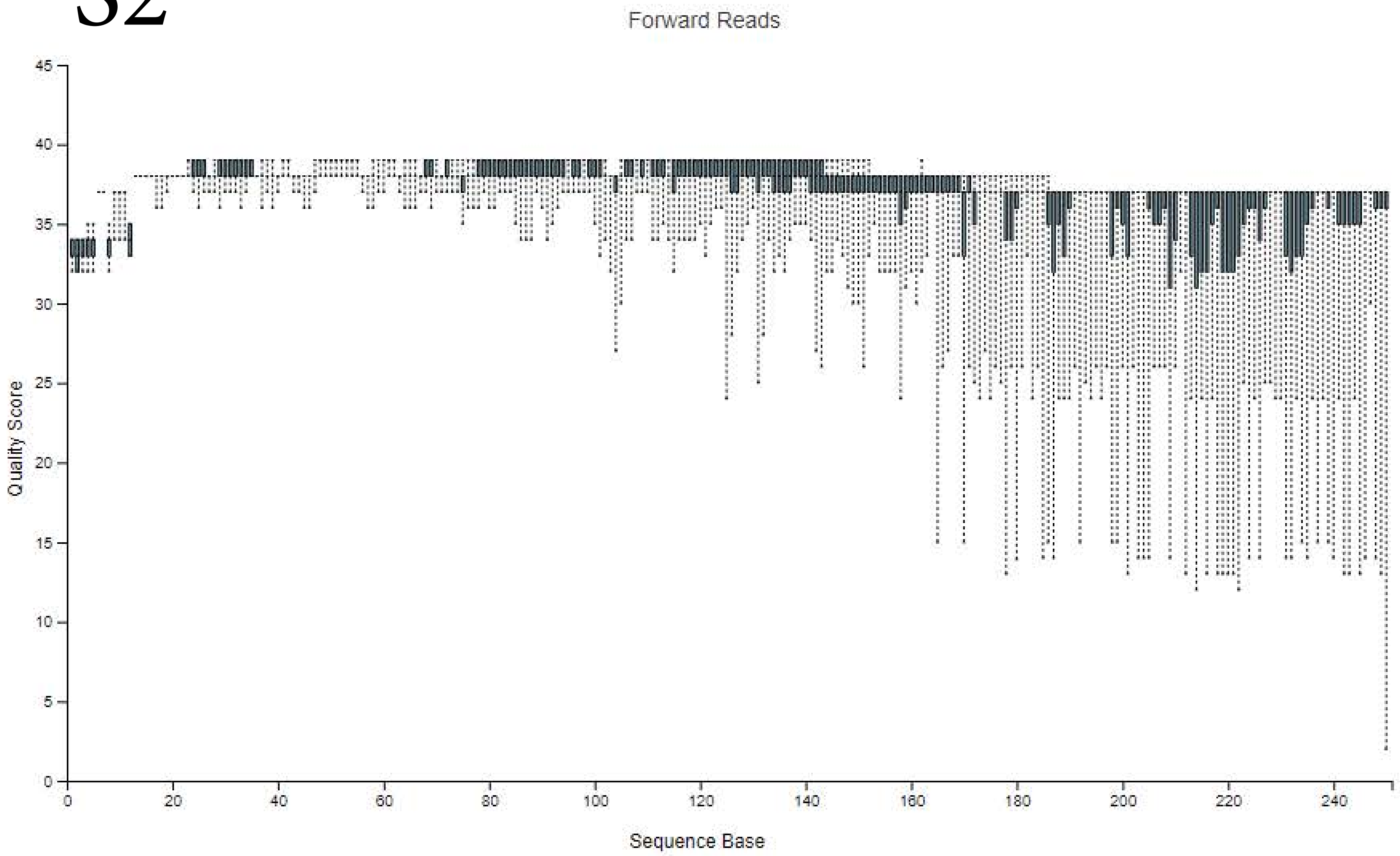

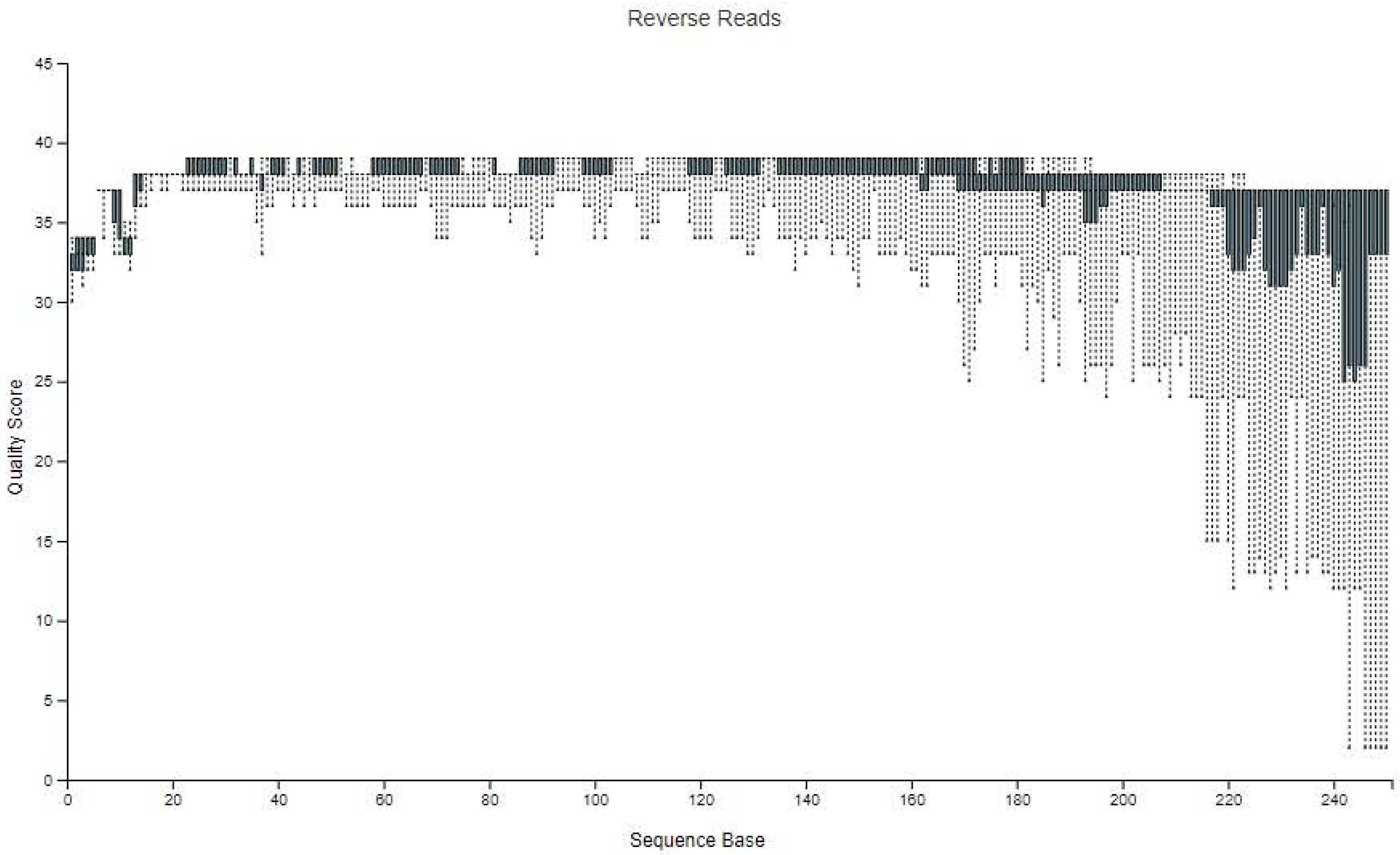

S3

Click and drag on plot to zoom in. Double click to zoom back out to full size. Hover over a box to see the parametric seven-number summary of the quality scores at the corresponding position.

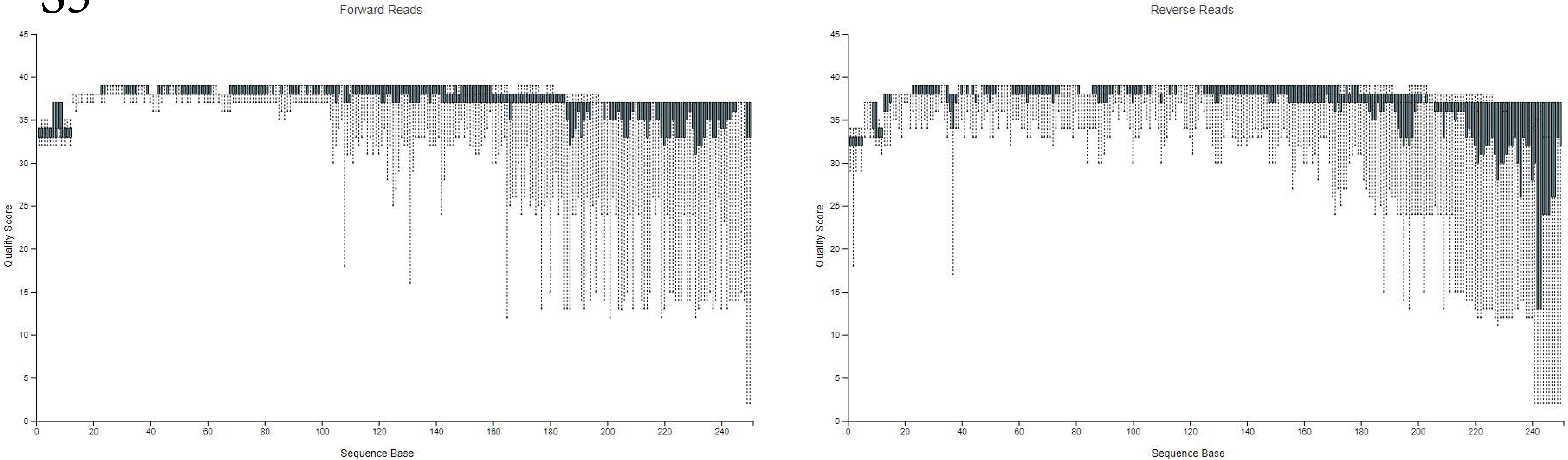

S4

Click and drag on plot to zoom in. Double click to zoom back out to full size. Hover over a box to see the parametric seven-number summary of the quality scores at the corresponding position.

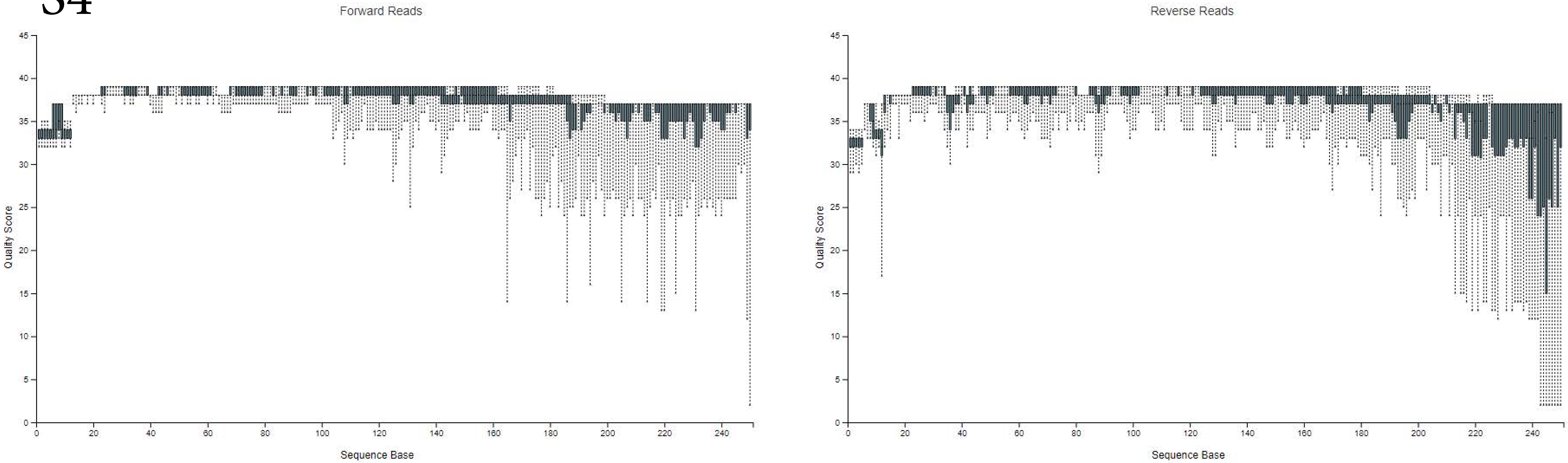

Supplement: Supplementary file 1 — Additional file 1. Figure S1. Phred quality plots of raw sequences for each mock community. [file 12859_2021_4410_MOESM1_ESM.pdf]
