# Supplementary material for: Concatenation of paired-end reads improves taxonomic classification of amplicons for profiling microbial communities
Source: BMC Bioinformatics. 2021 Oct 12;22:493. doi: 10.1186/s12859-021-04410-2 (PMC8507205; doi:10.1186/s12859-021-04410-2)
Supplement: Supplementary file 2 — Additional file 2. Figure S2. Precision, recall, and F-measure statistic comparisons averaged across mocks for each paired-end pipeline at different ASV percent abundance thresholds. Pipelines with taxonomy performed using SILVA had a higher F-measure mean at every threshold compared to Greengenes. Figure S3. ASV percent abundance threshold comparison of true positives (TPs), false positives (FPs), and false negatives (FNs) among paired-end pipelines using the SILVA references database. The unique number of genera identified for each of the ten mock communities per pipelines are summer together per pipeline per threshold. Genus counts do not consider ASV abundances. Figure S4. Heatmaps comparing balanced (G1, G2, S1) versus unbalanced (G3, G4, S2) mock communities with the same genera compositions. A) G1 vs G3, B) G2 vs G4, and C) S1 vs S2. Color scale shows proportional abundance of taxa within each mock / pipeline. Taxa are categorized by False Positives (blue) and True Positives (red). Relative abundance scale goes from dark blue (low) to red (high). [file 12859_2021_4410_MOESM2_ESM.pdf]

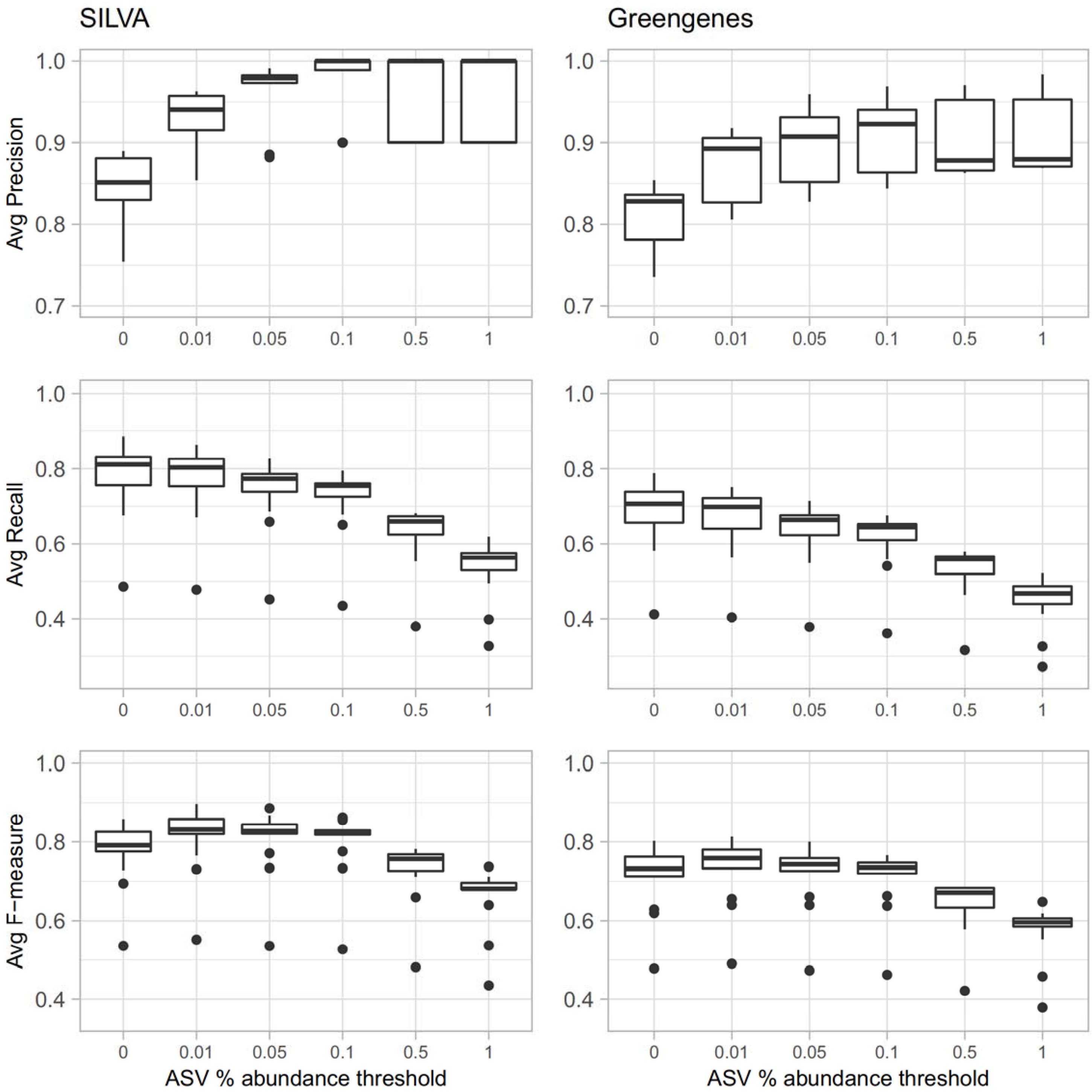

Supplementary Figure 2: Precision, recall, and F-measure statistic comparisons averaged across mocks for each paired-end pipeline at different ASV percent abundance thresholds. Pipelines with taxonomy performed using SILVA had a higher F-measure mean at every threshold compared to Greengenes.

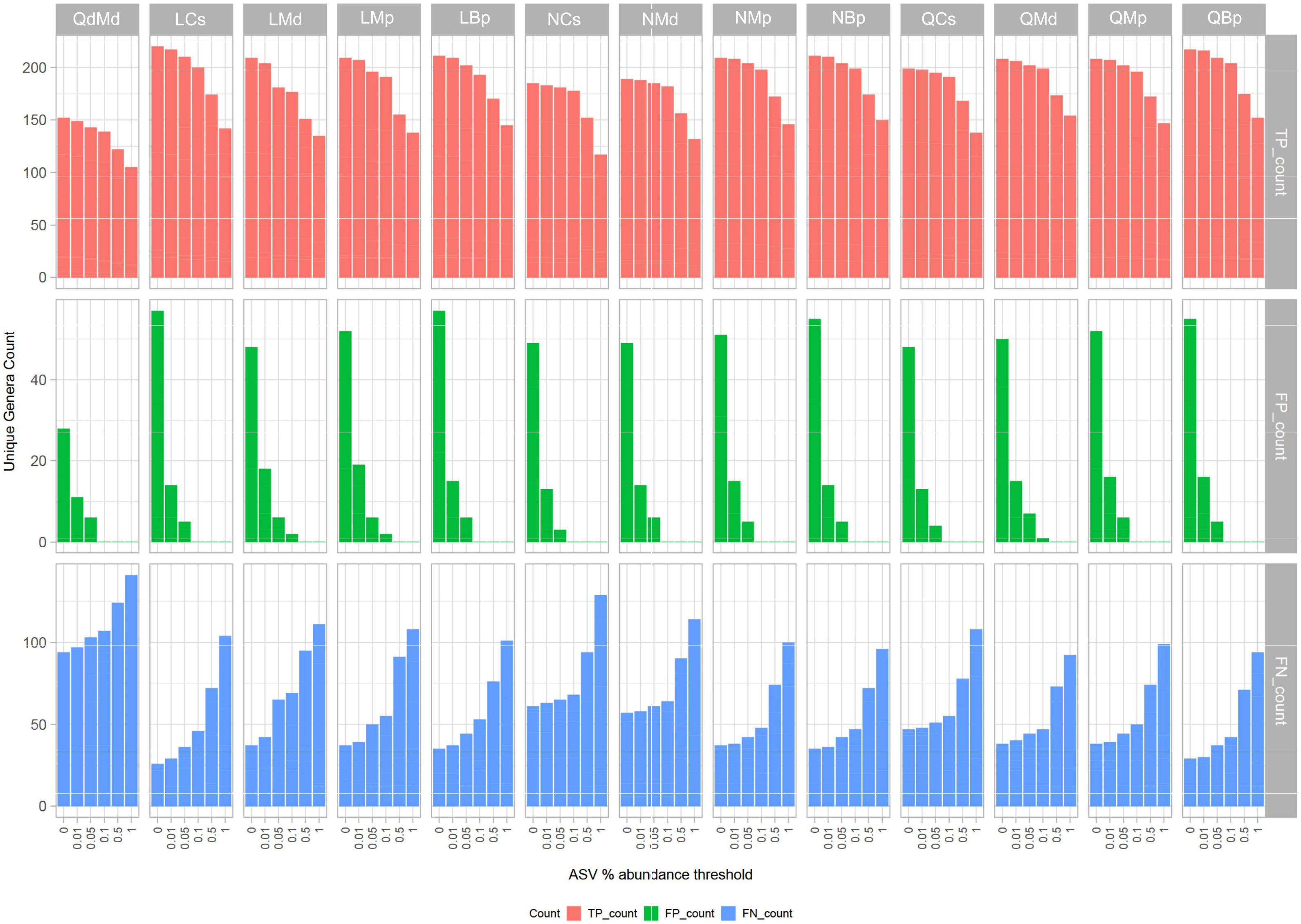

Supplementary Figure 3: ASV percent abundance threshold comparison of True Positives (TPs), False Positives (FPs), False Negatives (FNs) among paired-end pipelines using the SILVA reference database. The unique number of genera identified for each of the ten mock communities per pipeline are summed together per pipeline per threshold. Genus counts do not consider ASV abundances.

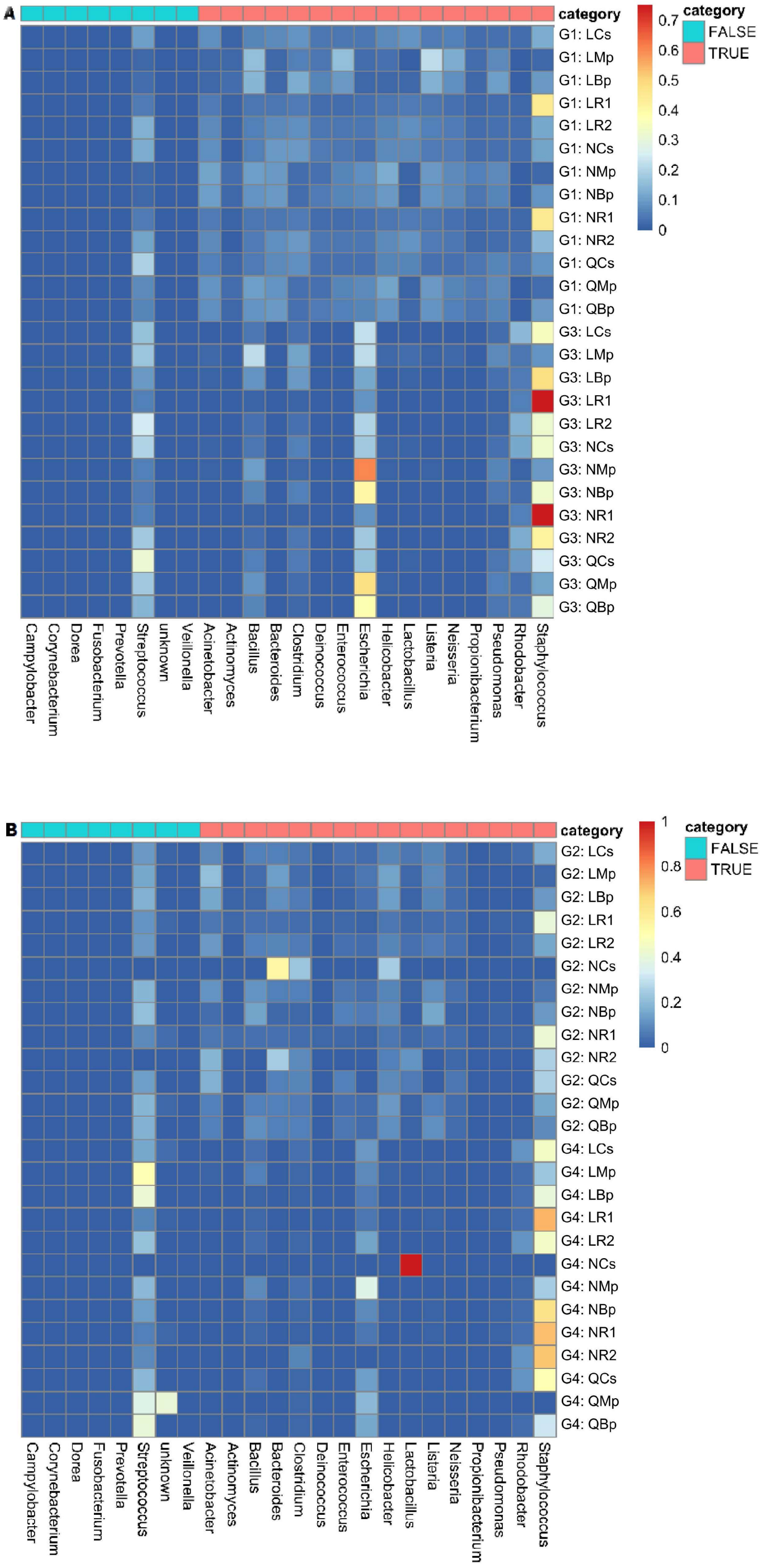
A
category
G1: LCs
G1: LMp
G1: LBp
G1: LR1
G1: LR2
G1: NCs
G1: NMp
G1: NBp
G1: NR1
G1: NR2
G1: QCs
G1: QMp
G1: QBp
G3: LCs
G3: LMp
G3: LBp
G3: LR1
G3: LR2
G3: NCs
G3: NMp
G3: NBp
G3: NR1
G3: NR2
G3: QCs
G3: QMp
G3: QBp
0.7
0.6
0.5
0.4
0.3
0.2
0.1
0
category
FALSE
TRUE
Campylobacter
Corynebacterium
Dorea
Fusobacterium
Prevotella
Streptococcus
unknown
Veillonella
Acinetobacter
Actinomyces
Bacillus
Bacteroides
Clostridium
Deinococcus
Enterococcus
Escherichia
Helicobacter
Lactobacillus
Listeria
Neisseria
Propionibacterium
Pseudomonas
Rhodobacter
Staphylococcus
B
category
G2: LCs
G2: LMp
G2: LBp
G2: LR1
G2: LR2
G2: NCs
G2: NMp
G2: NBp
G2: NR1
G2: NR2
G2: QCs
G2: QMp
G2: QBp
G4: LCs
G4: LMp
G4: LBp
G4: LR1
G4: LR2
G4: NCs
G4: NMp
G4: NBp
G4: NR1
G4: NR2
G4: QCs
G4: QMp
G4: QBp
1
0.8
0.6
0.4
0.2
0
category
FALSE
TRUE
Campylobacter
Corynebacterium
Dorea
Fusobacterium
Prevotella
Streptococcus
unknown
Veillonella
Acinetobacter
Actinomyces
Bacillus
Bacteroides
Clostridium
Deinococcus
Enterococcus
Escherichia
Helicobacter
Lactobacillus
Listeria
Neisseria
Propionibacterium
Pseudomonas
Rhodobacter
Staphylococcus

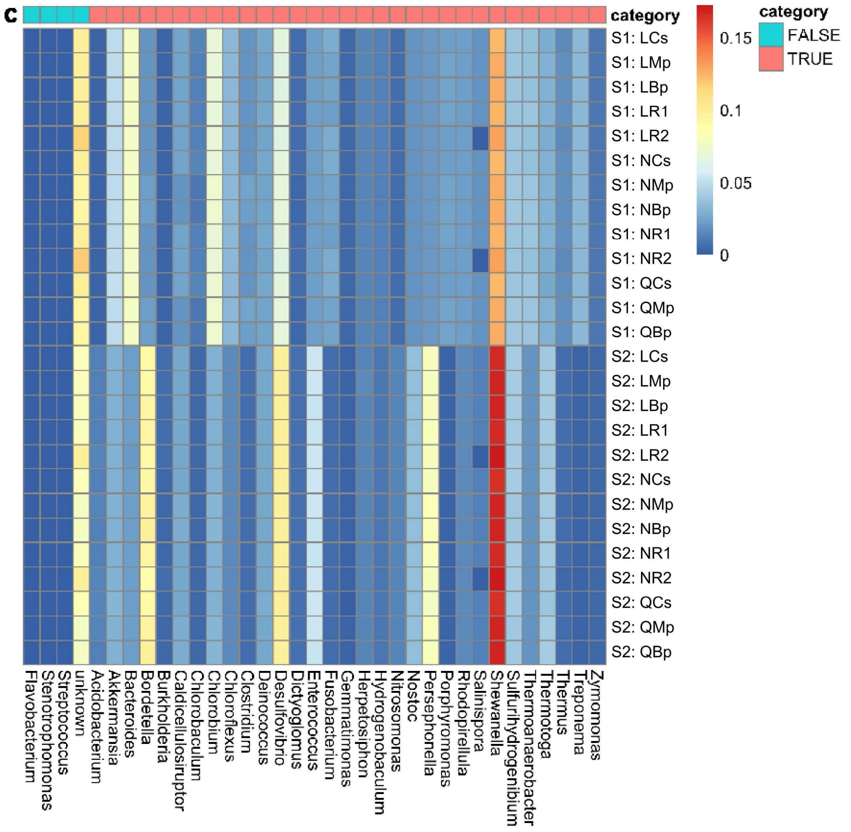

Supplementary Figure 4: Heatmaps comparing balanced (G1, G2, S1) versus unbalanced (G3, G4, S2) mock communities with the same genera compositions. A) G1 vs G3, B) G2 vs G4, and C) S1 vs S2. Color scale shows proportional abundance of taxa within each mock/ pipeline. Taxa are categorized by False Positives (blue) and True Positives (red). Relative abundance scale goes from dark blue (low) to red (high).
